# Variability in Tuberculosis Granuloma T Cell Responses Exists, but a Balance of Pro- and Anti-inflammatory Cytokines Is Associated with Sterilization

**DOI:** 10.1371/journal.ppat.1004603

**Published:** 2015-01-22

**Authors:** Hannah Priyadarshini Gideon, JiaYao Phuah, Amy J. Myers, Bryan D. Bryson, Mark A. Rodgers, M. Teresa Coleman, Pauline Maiello, Tara Rutledge, Simeone Marino, Sarah M. Fortune, Denise E. Kirschner, Philana Ling Lin, JoAnne L. Flynn

**Affiliations:** 1 Department of Microbiology and Molecular Genetics, University of Pittsburgh School of Medicine, Pittsburgh, Pennsylvania, United States of America; 2 Department of Immunology and Infectious Disease, Harvard School of Public Health, Boston, Massachusetts, United States of America; 3 Department of Radiology, PET Center, University of Pittsburgh Medical Center, Pittsburgh, Pennsylvania, United States of America; 4 Department of Pediatrics, Children’s Hospital of Pittsburgh of the University of Pittsburgh Medical Center, Pittsburgh, Pennsylvania, United States of America; 5 Department of Microbiology and Immunology, University of Michigan Medical School, Ann Arbor, Michigan, United States of America; Portland VA Medical Center, Oregon Health and Science University, UNITED STATES

## Abstract

Lung granulomas are the pathologic hallmark of tuberculosis (TB). T cells are a major cellular component of TB lung granulomas and are known to play an important role in containment of *Mycobacterium tuberculosis* (Mtb) infection. We used cynomolgus macaques, a non-human primate model that recapitulates human TB with clinically active disease, latent infection or early infection, to understand functional characteristics and dynamics of T cells in individual granulomas. We sought to correlate T cell cytokine response and bacterial burden of each granuloma, as well as granuloma and systemic responses in individual animals. Our results support that each granuloma within an individual host is independent with respect to total cell numbers, proportion of T cells, pattern of cytokine response, and bacterial burden. The spectrum of these components overlaps greatly amongst animals with different clinical status, indicating that a diversity of granulomas exists within an individual host. On average only about 8% of T cells from granulomas respond with cytokine production after stimulation with Mtb specific antigens, and few “multi-functional” T cells were observed. However, granulomas were found to be “multi-functional” with respect to the combinations of functional T cells that were identified among lesions from individual animals. Although the responses generally overlapped, sterile granulomas had modestly higher frequencies of T cells making IL-17, TNF and any of T-1 (IFN-γ, IL-2, or TNF) and/or T-17 (IL-17) cytokines than non-sterile granulomas. An inverse correlation was observed between bacterial burden with TNF and T-1/T-17 responses in individual granulomas, and a combinatorial analysis of pair-wise cytokine responses indicated that granulomas with T cells producing both pro- and anti-inflammatory cytokines (*e.g.* IL-10 and IL-17) were associated with clearance of Mtb. Preliminary evaluation suggests that systemic responses in the blood do not accurately reflect local T cell responses within granulomas.

## Author Summary

The characteristic feature of *Mycobacterium tuberculosis* (Mtb) infection is the formation of lesions, which are organized structures of immune cells in the lungs called granulomas, which contain the bacteria. When the granuloma functions effectively, it can kill the bacteria. T cells (a type of immune cell, also present in granulomas) are known to play an important role in control of tuberculosis. However, functions of T cells at individual granuloma levels are unknown. Here, we studied the functional characteristics of T cells, which are defined by the production of chemical messengers (cytokines) at the granuloma level in a non-human primate model. We compared the relationship between cytokine response and the number of bacteria (Mtb) in each granuloma. Each granuloma was found to be unique, suggesting different types exist within an animal. Only a small proportion of T cells produced any cytokine, but different types of cytokines were observed within each granuloma. A balance between different types of cytokine was associated with more killing of bacteria in granulomas. Understanding how to improve the T cell responses to obtain killing of bacteria in the granuloma will be important for vaccine development.

## Introduction

*Mycobacterium tuberculosis* (Mtb) remains a major threat to global health. The latest World Health Organization analysis of the global burden of tuberculosis (TB) estimates 8.7 million new cases, 9.6–13.0 million prevalent cases, and 1.4 million deaths per year in 2011 [1]. However, only 5–10% of those infected with Mtb will develop active disease over their lifetime, while the other ∼90% remains asymptomatic (referred to as “latent” infection) with a 5–10% chance of reactivation over their lifetime. Thus, it is clear that the human immune response is quite capable of controlling Mtb infection.

Mtb infection is characterized by the formation of granulomas, usually in the lungs and lymph nodes [2]. The tuberculous granuloma is an organized structure of immune cells that forms in response to persistent Mtb infection, and consists of macrophages, neutrophils, and lymphocytes [3–6]. Granulomas function both as the niche in which bacilli can grow or persist and an immunological microenvironment in which host cells interact to control and prevent dissemination. The mere presence of granulomas is insufficient to control infection; instead, proper functioning of all granulomas in a host determines the ultimate outcome of infection [4]. T lymphocytes are considered critical to control of initial and persistent Mtb infection, mediating the inflammatory balance suggested in histological and flow cytometry studies [7–12]. Important roles for T cell produced cytokines (IFN-γ, IL-2, TNF, IL-17 and IL-10) have been demonstrated in animal model studies, with a subset of these cytokines demonstrated to be critical in humans as well [12–22]. However, in humans, responses are generally measured in blood so very little is known about T cell function at the level of the granuloma.

To study and understand the functions of T cells in granulomas requires obtaining fresh lung tissue containing granulomas, which is very difficult in humans. Therefore, an animal model that recapitulates human disease and pathology is necessary to understand the role of adaptive immune response in tuberculosis. The standard murine models, although useful for investigating immune responses and pathogenesis, do not establish latent infection and form granulomatous infiltrations rather than organized granulomas [23]. However, non-human primates, primarily macaques, are remarkably similar to humans in terms of infection outcome and presentation as well as pathology, and have the additional advantage of research reagents that permit the analysis of immunological components in controlling the disease. Cynomolgus macaques develop clinically active or latent infection with granulomas that are extremely similar to those in human TB [24–27]. We previously reported that a spectrum of lesions are found in individual animals and among animals with active or latent infection [25], as has been described for humans [28]. In addition, recent studies from our group support that progressive and healed lesions can coexist within the same animal, with nearly all animals capable of sterilizing at least a subset of individual granulomas. However, animals with active TB present with a subset of lesions that do not control infection which results in dissemination of the disease [29].

Taken together, available data support the hypothesis that the outcome of Mtb infection is determined at a local, not a systemic level. What controls infection at a local level is unknown, but is likely a combination of cellular and cytokine mediators induced by the bacilli together with physiological constraints [2]. The functionality of T cells, i.e., cytokine responses, and their correlation with bacterial burden in individual granulomas within an animal has not been investigated. Therefore, to understand the nature of individual tuberculosis granulomas within an animal with respect to T cells, cytokine profile and bacterial burden, we evaluated functional characteristics of T cell pro-inflammatory (IFN-γ, IL-2, TNF and IL-17) and anti-inflammatory or regulatory (IL-10) cytokine responses in cynomolgus macaques infected with Mtb. Our previously published studies on bacterial burden in granulomas and PET/CT and histology showed that a spectrum of granulomas exist within a host [2,25,29,30], with extensive overlap among animals of different clinical states. Here we extend our evaluation of granulomas to focus on T cell cytokine profiles associated with the ability of individual granulomas to control bacterial burden (sterilizing and non-sterilizing) initially without regard to whether the host developed active disease or remained latently infected. Finally, in a preliminary analysis, we compared local T cell cytokine responses from granulomas of each animal to those of the systemic T cell cytokine response to determine how accurately the blood can be used as a “read-out” for local immune responses.

## Results

### Study population

Thirty-four NHP infected with a low dose of Mtb were necropsied primarily as controls for other studies [2,30] and were included in this study. Twenty-eight animals were necropsied >17 weeks of Mtb infection (median 313, range 124–601 days), which included 13 animals with clinically active disease and 15 animals with clinically latent infection [25]. Median time to necropsy post infection was 222 days (124–400 days) for active animals and 367 days (284–601 days) for latent animals. The animals were classified based on clinical signs and microbiologic cultures, as previously described [24,25]. In addition, six animals necropsied at approximately 11 weeks post infection (median 77.5 days, 72–85 days) as controls were also included in this analysis.

**Figure 1:**
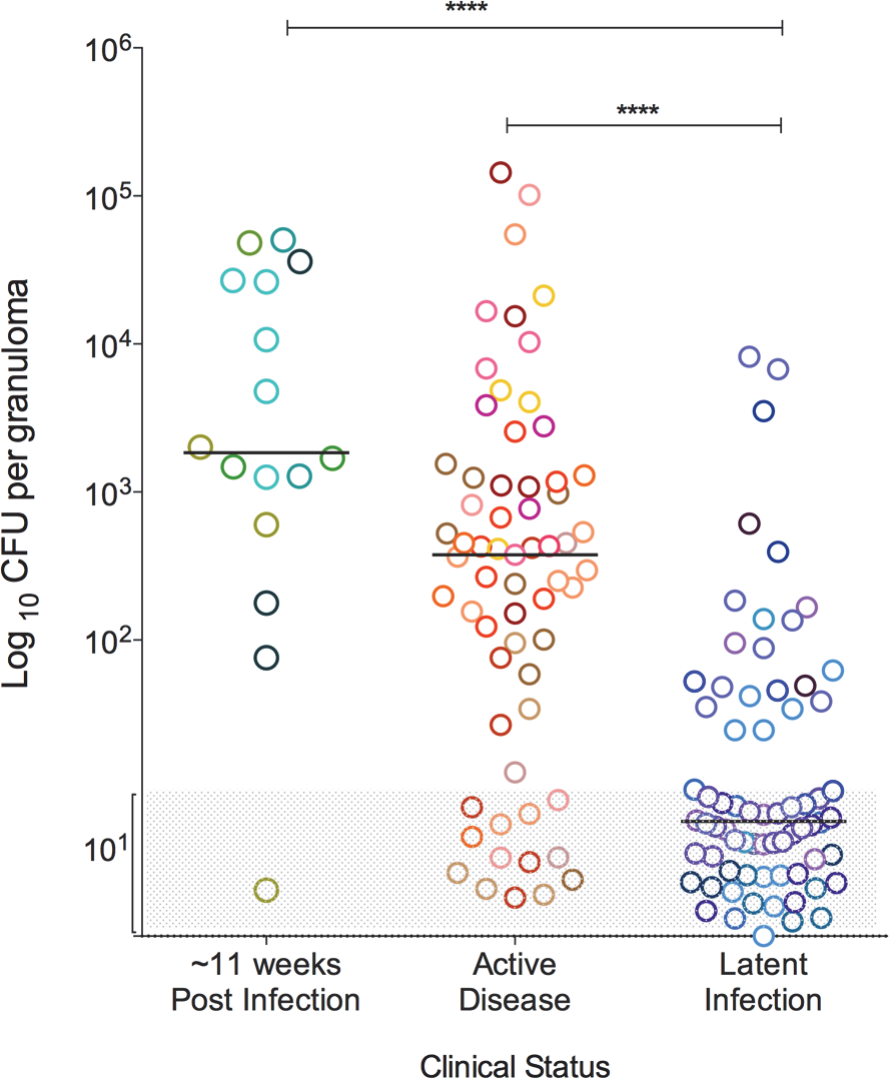
Bacterial burden of individual granulomas. Bacterial burden is represented as the colony forming units (CFU) in logarithmic scale, per granuloma. Greyed area represents sterile granulomas (indicated as log_10_ 1), which were assigned arbitrary range of values so that all granulomas can be seen in the graph. Each symbol represents a granuloma and a color represents granulomas from one animal. Granulomas are grouped as per the clinical status of the animal, where those animals that were infected for ∼11 weeks are in shades of green, active disease in shades of warm colors and latent infection in shades of cool colors. (****: p<0.0001, Dunn’s multiple test comparison).

Granulomas were observed in all animals following Mtb infection with substantial variability in types and numbers of granulomas. As previously reported [2], in the animals used for this study, the number of culturable Mtb bacilli from individual granulomas covered a large range in individual animals, from 0 (sterilized) to ∼10^6^ CFU/granuloma (S1 Fig.). The median CFU/granuloma was highest in animals infected for ∼11 weeks (log_10_ 3.492, IQR 2.646–4.461) and those with active disease (log_10_ 2.6, IQR: 1.5–3.1), compared to those from animals with latent infection (median log_10_ 1, IQR: 1–1.6) (p<0.0001) (Fig. 1). However, the CFU/granuloma overlapped substantially among the clinical groups, with a significant fraction of sterile granulomas even in animals with active TB. These are in line with data previously published from our lab [2]. Since granuloma bacterial burden is variable within individual animals as well as across animals from different clinical classifications, our analysis primarily focused on the evaluation of granuloma T cell profiles based on bacterial burden without regard to clinical classification. This study only focused on individual granulomas as defined by PET/CT imaging obtained prior to necropsy [29] and at necropsy by the pathologist. Thus, complex pathologies as seen in active TB such as coalescing granulomas, consolidations, or TB pneumonia, were excluded.

### Numbers of T cells in the granuloma vary within and among animals

**Figure 2:**
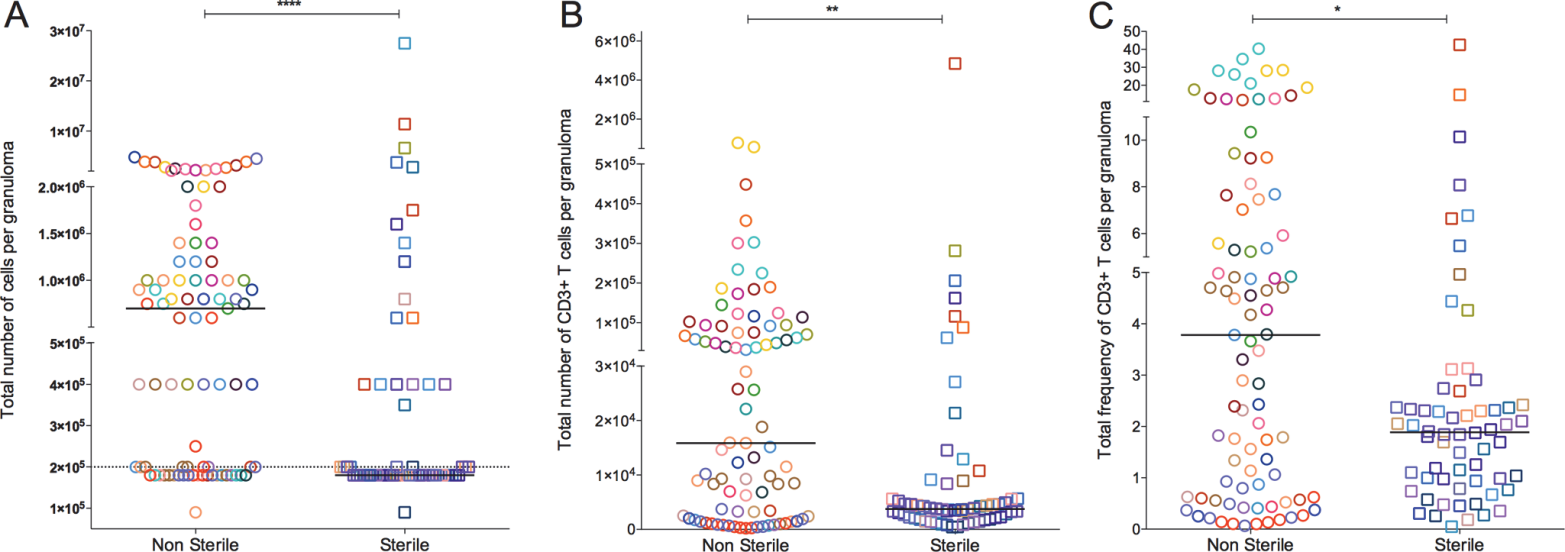
Absolute cell counts per granuloma. **A:** Total number of cells obtained from granuloma. Granulomas with a cell count less than the detection limit was assigned 9×10^4^ before the correcting for the dilution factor. **B**: Total number of T cell counts, defined by CD3+ per granuloma. **C** is the total percentage of live CD3+ cell per granulomas and was used to extrapolate the absolute T cell count per granuloma (B) from the total granuloma cell count (A). Each symbol represents a granuloma. Each color represents an animal. Granulomas are grouped based on bacterial burden of the granuloma: non-sterile (Mtb cultured, in open circles) or sterile (in open squares). Solid line indicates median in each group. Dotted line indicates the detection limit (1×10^5^) before correcting for the dilution factor. (****: p<0.0001, **: p≤0.001, Non-parametric Mann-Whitney).

Total cell numbers varied amongst individual granulomas. Overall, the median cell count of all granulomas (by counting live cells in homogenates using trypan blue exclusion prior to further manipulations) was 4×10^5^ (IQR 1.8×10^4^–1×10^6^). The total cell count of granulomas from within an individual animal also varied. Non-sterile granulomas had significantly higher cell counts than sterile granulomas (Fig. 2A). Similarly, numbers and frequency of live CD3+ T cells were variable among granulomas within individual animals. Non-sterile granulomas had significantly higher T cell counts (p = 0.0011) (Fig. 2B) and frequency of live CD3+ T cells (p = 0.0172) (Fig. 2C) when compared to sterile granulomas. Due to the limited number of viable T cells obtained from many of the granulomas, our analyses of cytokine production were restricted to the CD3+ T cell population. Total cell count, T cell numbers, and bacterial burden correlate with the size of the granuloma (S1 Table).

### Limited numbers of T cells producing cytokines from individual granulomas

It is generally accepted that Th1-type T cell responses (characterized by the cytokines IFN-γ, IL-2, or TNF) are important in control of Mtb infection [10,12,18,20,31,32]. Th17 CD4+ T cells (producing IL-17) have also been implicated in control of this infection, particularly in the early phases [7,33,34]. These pro-inflammatory responses are considered to be necessary for activating macrophages to kill Mtb and organizing cell recruitment to the granuloma. On the other hand, the importance of anti-inflammatory (regulatory) responses, such as T cells producing IL-10, in the granuloma is more controversial [35]. In fact, little data exist regarding the types of T cell responses in individual granulomas in humans or in a model with substantial similarity to humans [36–38]. Thus, we investigated the patterns of pro-inflammatory (T-1 and T-17: with reference to CD3+ T cells producing Th-1/Th-17 type cytokines) and anti-inflammatory/regulatory (IL-10) T cell cytokine production in individual granulomas following stimulation with peptides from Mtb-specific RD-1 encoded proteins ESAT-6 and CFP-10.

**Figure 3:**
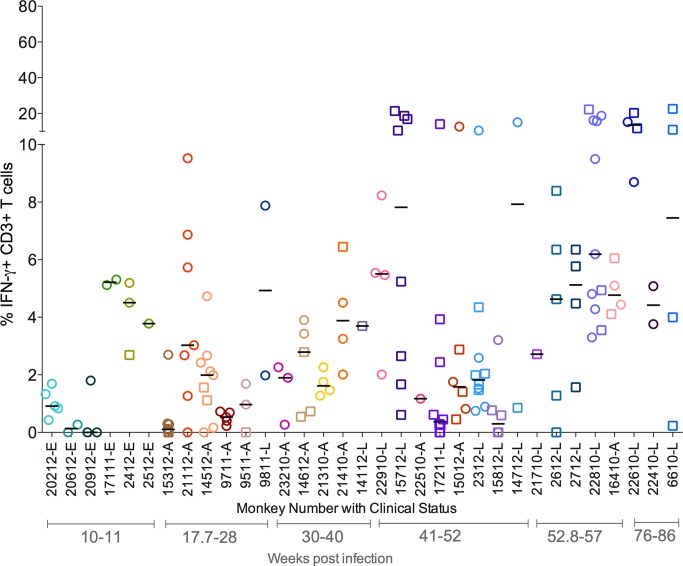
Proportion of T cells with IFN-γ response from each animal included in the study. Each symbol represents an individual granuloma. Each individual granuloma had a distinct cytokine profile and there was a range of cytokine response in each animal. Similar responses were seen for other cytokines (S2 Fig.). Each symbol represents a granuloma. Each color represents an animal. Granulomas are marked based on bacterial burden of the granuloma: non-sterile (Mtb cultured, in open circles) or sterile (in open squares). Clinical status is represented for each animal along with monkey number [“E”: ∼11 weeks post infection; “A”: Active disease; “L”: Latent Infection]. Animals are arranged in the order of increasing post-Mtb infection time.

As with bacterial and T cell numbers, individual granulomas, even within a single animal, displayed highly distinct and variable cytokine profiles. In most animals, the range of T cell cytokine responses was noted to be approximately 1–8% with occasional outlier granulomas that either did not mount any detectable cytokine response or had a higher frequency of T cells making cytokines. For instance, IFN-γ responses were very low or undetectable in all granulomas obtained from 4 animals (20612, 9711,15312 and 17211), while in others the range of IFN-γ responses was from <5 to 15%, with granulomas from a subset of animals giving responses over 15% (Fig. 3). This pattern was observed for all single cytokines tested, with the majority of granulomas having low frequencies of T cells producing T-1 or T-17 cytokines (S2A–S2E Fig.). When all granulomas are analyzed across all animals, the average frequency of T cells making any of the cytokines measured was ∼8%, a surprisingly low frequency given the importance of T cells in control of this infection.

**Figure 4:**
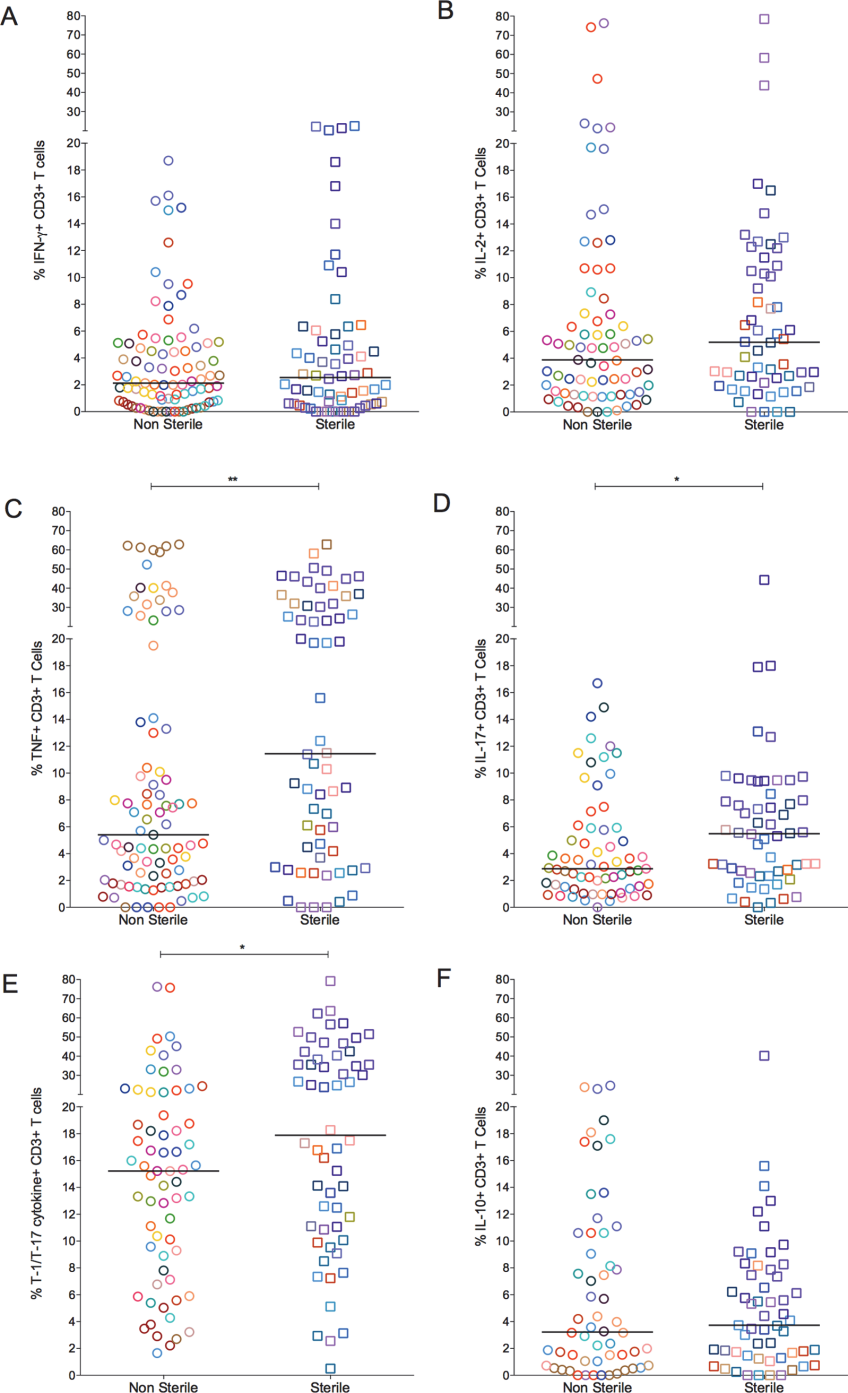
The proportion of T cells with cytokine response of IFN-γ (A), IL-2 (B), TNF (C), IL-17 (D), T-1/T-17 (E) and IL-10 (F). Each symbol represents a granuloma. Each color represents an animal. Granulomas are marked and grouped based on bacterial burden of the granuloma: non-sterile in open circles and sterile in open squares. The frequency of cytokine producing T cells overlapped considerably between the groups (* = <0.05; **<0.01, Nonparametric Mann-Whitney test).

As a spectrum of granuloma T cell cytokine profile was noted even within an individual host, we compared T cell responses in granulomas with or without culturable bacteria. The frequency of cytokine producing T cells overlapped considerably across both sterile and non-sterile granulomas. Nevertheless, sterile granulomas had modestly higher TNF (p = 0.0091), IL-17 (p = 0.0344) and T-1/T-17 (CD3+ T cells producing single or combinations of IFN-γ, IL-2, TNF, or IL-17 cytokines) (p = 0.0273) cytokine producing T cells than non-sterile granulomas. This finding is independent of the clinical classification of animals from which sterile or non-sterile granulomas were obtained (Fig. 4).

### T cell exhaustion does not appear to account for the low frequencies of cytokine producing T cells in most granulomas

In chronic infections, T cell exhaustion can contribute to a reduced ability to produce cytokines [39–41]. To explore this as a possible contributor to the low frequency of T cell responses in granulomas, a subset of granulomas was stimulated with the non-specific stimulators PDBu and ionomycin and cytokine responses were measured. In response to PDBu and ionomycin, higher frequencies of T cells from individual granulomas produced IFN-γ (median 27.25%, IQR 8.7%–47.40%) and TNF (32.25%, 16.23%–40.85%), with modestly higher frequencies of IL-2 (5.67%, 3.5%–12.43%) producing cells and no change in IL-17 (3.8%, 2.3%–6.49%) production. When examining combined cytokine responses (i.e. ability to produce any of the T-1/T-17 cytokines) in response to non-specific stimulation, ∼50% of granuloma T cells on average produced cytokines (S3 Fig.). This is much higher than stimulation with ESAT-6/CFP-10, suggesting that in most granulomas, the T cells are capable of producing cytokines. However, there was a subset of granulomas that still gave very low responses, and these may be granulomas in which the majority of T cells are not capable of responding to stimulation or are exhausted.

In a subset of granulomas, we evaluated the expression of exhaustion markers CTLA-4 and PD-1 on T cells. Although overall frequencies of cells positive for these markers were low (S4 Fig.), CTLA-4^−^/PD-1^+^ were most common, while CTLA-4^+^/PD-1^−^ or CTLA-4^+^/PD-1^+^ were only seen at very low frequency in granulomas (S4A Fig.). Next, we explored the cytokine production capacity in T cells expressing these markers, following stimulation with Mtb-specific RD-1 antigens (S4B–S4F Fig.). Overall, the frequency of cytokine producing T cells that also expressed CTLA-4 or PD-1 or both was very low, with the highest being 2.4% of IL-10 producing T cells co-expressing PD-1 (S4F Fig.). CTLA-4^−^/PD-1^+^ T cells generally had slightly higher cytokine responses than those expressing only CTLA-4 or both CTLA-4 and PD1 (S4B–S4F Fig.). Taken together these data suggest that the limited T cell response observed in the T cell of granulomas is not solely due to the exhaustion of T cells.

### Single cytokine producing T cells are predominant in lung granulomas

**Figure 5:**
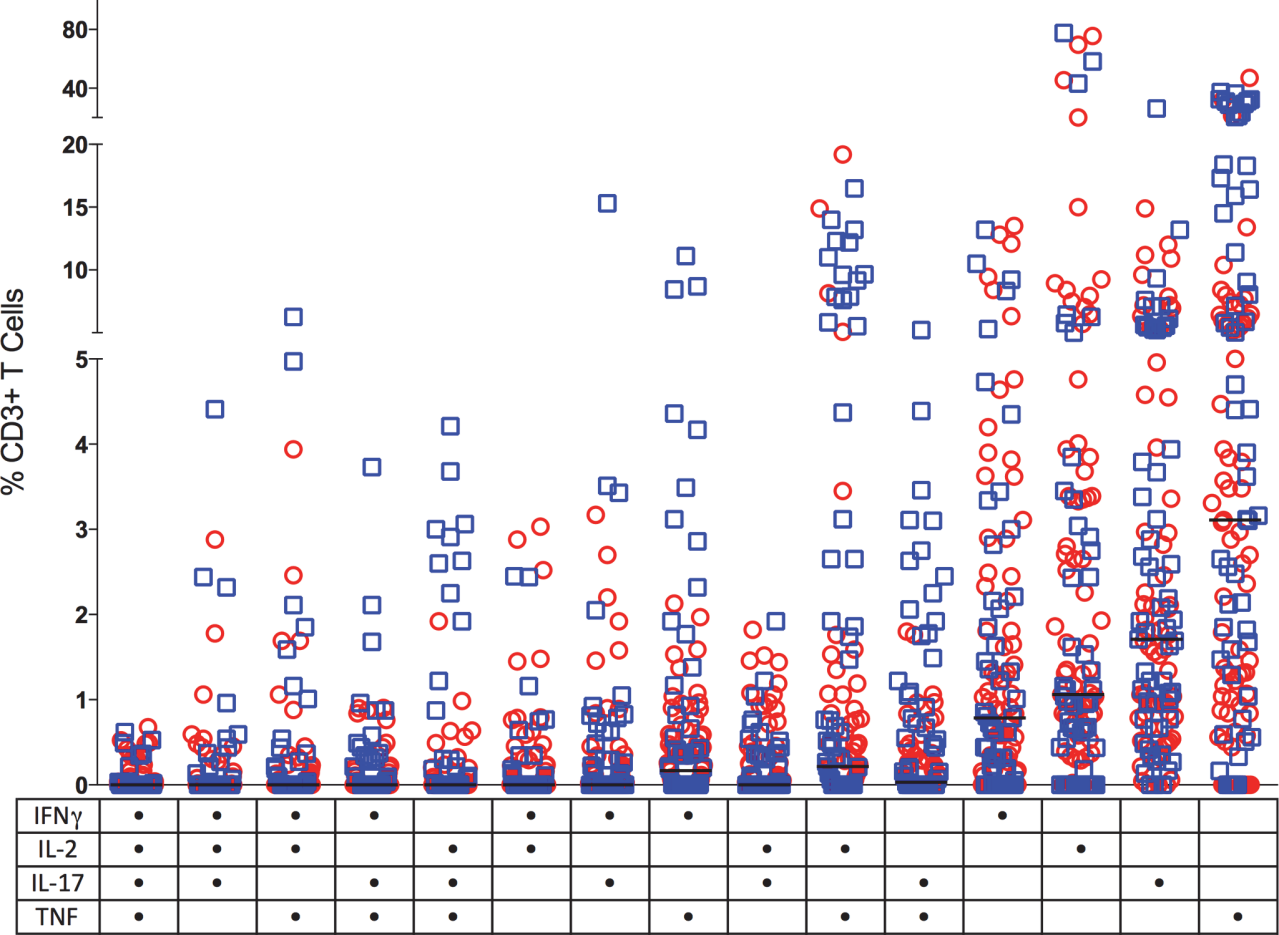
CD3+ T cells making single (one) or multiple (two, three or four) cytokines from individual granulomas. Non-sterile granulomas are in open circles (red) and sterile granulomas are represented as open squares (blue). Solid line indicates median response. The table on the X axis reflects the groups positive for each cytokine represented by a dot. There was no significant difference between the multiple cytokine pattern of response between sterile and non-sterile granulomas or between various clinical states of the animal. (n = 121).

Multifunctional T cells (those producing several cytokines) have been suggested to be important in the control of infections, although there are conflicting data on the protective capacity of these cells in tuberculosis [42–46]. Most human TB studies on multifunctional T cells have focused on cells derived from the periphery (e.g. blood), where the frequency of multifunctional T cells is often, although not always, associated with active disease rather than protection [44,47,48]. We investigated whether cytokine producing T cells in granulomas are multifunctional by assessing the ability to produce 1–5 cytokines. IL-10 is not usually considered to be produced by T cells making other pro-inflammatory cytokines, although in a minor subset of granulomas examined, there was a small population of T cells (1.2%) that made both IL-10 and IL-17. Interestingly, T cells with this phenotype have been associated with control of some bacterial infections rather than with autoimmune disease [49,50]. Thus, we excluded IL-10 from the analysis of multifunctional T cells. Surprisingly, T cells producing single cytokines were most frequently observed in the granulomas (Fig. 5), particularly TNF and IL-17. There were T cells present that were producing both TNF and IL-2 in a subset of granulomas. However, very few granulomas had T cells that were classically multi-functional (Fig. 5). There was no significant difference in the multiple cytokine responses between sterile and non-sterile lesions and amongst different clinical groups.

### Balance between pro- and anti-inflammatory cytokine producing T cells in lung granuloma

To investigate the relationships of functional T cell populations within individual granulomas, we assessed correlations among cytokine responses. The cytokine balance may ultimately determine control of bacterial burden in the granuloma. Overall, there was a significant direct correlation between each of the T-1 pro-inflammatory cytokines (IFN-γ, IL-2 and TNF) and among T-1 and T-17 cytokines (i.e., in individual granulomas, higher responses of cytokine X were observed with higher responses of cytokine Y) (Table 1). Thus, even though there are few T cells that produce multiple cytokines, T cells producing individual T-1 and T-17 cytokines tend to be present together in the same granulomas, suggesting that granulomas are “multi-functional”. Surprisingly, there was also a direct correlation of T-1 and T-17 cells with T cells producing IL-10 in individual granulomas (Table 1). The correlation between IL-17 and IL-10 producing T cells was significant in both non-sterile and sterile granulomas. In addition, sterile granuloma had a significant direct correlation between IL-10 and IFN-γ and total T cells producing T-1 or T-17 cytokines (Table 1), suggesting that a balance between pro and anti-inflammatory cytokines are required for reduced pathology and control of bacterial burden.

**Table 1:** Pairwise correlation of cytokine levels within granulomas based on bacterial burden.

**Cytokine variables**	**Non sterile Granulomas**		**Sterile Granulomas**	
	**Spearman ρ**	**Prob>|ρ|**	**Spearman ρ**	**Prob>|ρ|**
IL-2 vs IFN-γ	**0.4269**	**0.0002**	−0.1082	0.4272
TNF vs IFN-γ	0.0847	0.4356	−0.1047	0.4181
TNF vs IL-2	**0.3259**	**0.0049**	**0.3912**	**0.0029**
TNF vs IL-10	−0.0789	0.5631	0.236	0.0828
TNF vs IL-17	0.1122	0.3736	**0.5186**	**<.0001**
IL-17 vs IFN-γ	**0.2604**	**0.0362**	**0.298**	**0.0257**
IL-17 vs IL-2	0.2389	0.0553	**0.462**	**0.0003**
IL-17 vs IL-10	**0.563**	**0.0005**	**0.7082**	**<.0001**
IL-10 vs IFN-γ	0.2236	0.0976	**0.3722**	**0.0051**
IL-10 vs IL-2	0.0022	0.989	0.2155	0.1369
T-1/T-17 vs IL-10	0.0014	0.9938	**0.3791**	**0.0072**

### Pro-inflammatory cytokine levels are inversely correlated with bacterial burden

**Figure 6:**
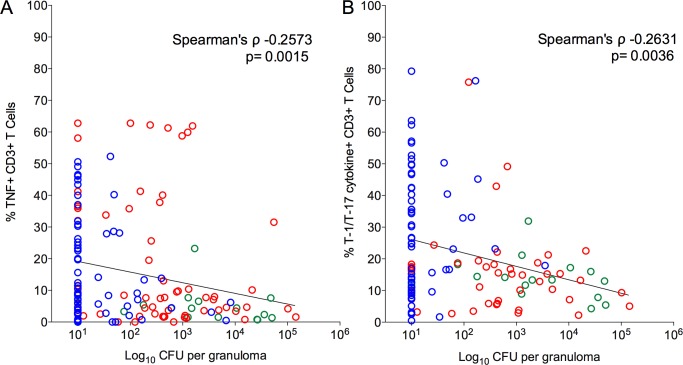
Correlation of pro-inflammatory T cell cytokine response to the bacterial burden (CFU per granuloma) in each of the individual granulomas. Significant inverse correlation was observed between frequencies of TNF (**A**) and T-1/T-17 (**B**) producing T cells and bacterial burden. CFU per granuloma value of zero were transformed to “1” and included in this analysis. Each symbol represents a granuloma. Line indicates the slope. The clinical status of an animal is represented by the color of the granuloma. Animals with active disease are in red, latent infection in blue and those with ∼11 weeks of infection are in green.

We next explored whether T cell cytokine responses were related to bacterial burden in individual lesions. Overall, a significant inverse correlation was observed between T-1/T-17 and TNF cytokine producing T cells and bacterial burden (CFU per granuloma), i.e., higher frequencies of cytokine responses were related to lower the bacterial burden (Fig. 6, Table 2). This suggests that a pro-inflammatory cytokine environment associates with decreased bacterial burden, most likely by stimulating cells to kill bacilli in the granuloma. When only non-sterile granulomas were analyzed, there was a significant inverse correlation between bacterial burden and IFN-γ and T-1/T-17 cytokine producing T cells, further supporting that higher pro-inflammatory cytokines are associated with control of bacterial burden (Table 2).

**Table 2:** Correlation between granuloma bacterial burden and T cell cytokine response.

**Variables**		**All Animals**		**Non-Sterile granulomas**	
		**Spearman ρ**	**Prob>|ρ|**	**Spearman ρ**	**Prob>|ρ|**
**Log_10_ CFU per granuloma**	IFN-γ	−0.112	0.1739	**−0.2632**	**0.0138**
	IL-2	−0.1622	0.0663	−0.1849	0.1173
	TNF	**−0.2573**	**0.0015**	−0.1922	0.0746
	IL-17	−0.1617	0.0765	0.0658	0.6025
	IL-10	0.0155	0.8714	0.0223	0.8703
	T-1/T-17	**−0.2631**	**0.0036**	**−0.2776**	**0.0252**

### Combination of pro- and anti- inflammatory cytokines are associated with sterilization of granulomas

**Figure 7:**
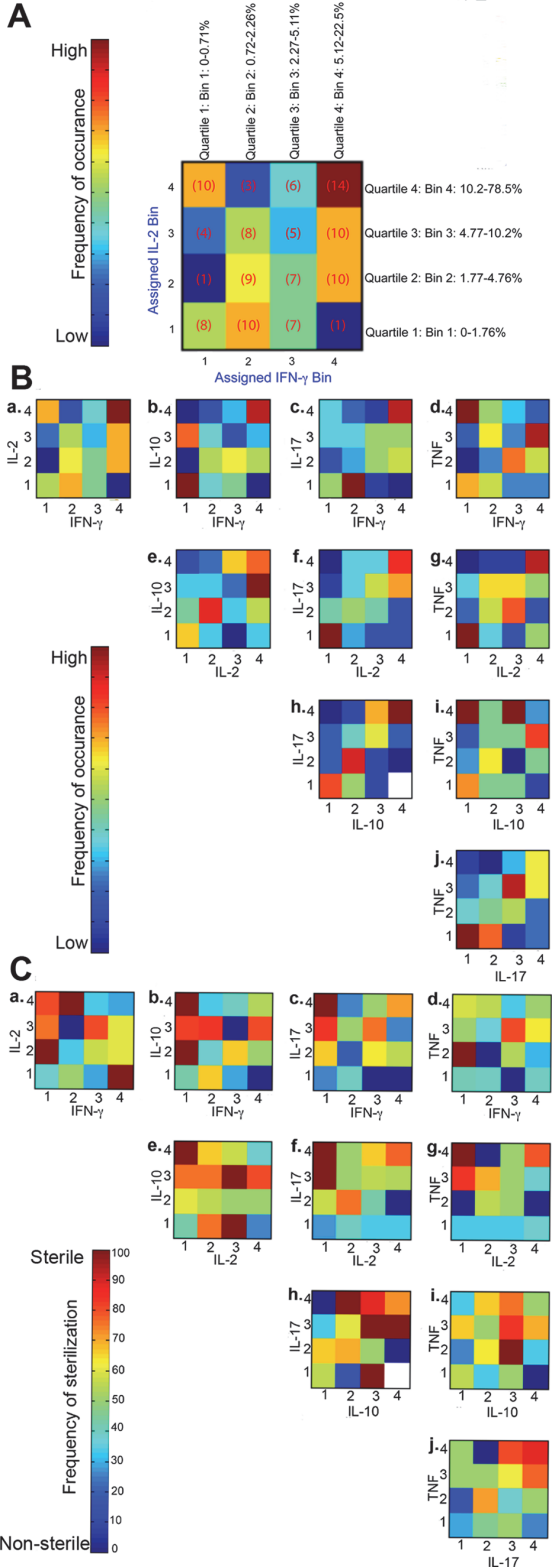
Pairwise occurrence matrix and sterilization frequency of T cell cytokines. **A** is an example figure: For each cytokine variable of the granuloma, continuous percentage values were binned into one of four categories depending on quartile distribution (bins 1, 2, 3, or 4). Number of granulomas (number of times particular combination of binned cytokines occurred) in each of the bins were counted, for example, low IFN-γ (bin 1) and high IL-2 (bin 4) had 10 granulomas and summarized in a 4×4 co-occurrence matrix. For each pairwise cytokine comparison, the frequency of occurrence was summarized in heat-map form. **B** is the frequency of occurrence for all pairwise combination of T cell cytokines from individual granulomas. The scale represents the frequency of occurrence ranging from none/low to high. **C** is the sterilization frequency where matrices were constructed in a similar fashion by calculating the number of times sterilization occurred in a given particular cytokine combination and magnitude. Each square in each 4×4 heat-map therefore represents the frequency of sterilization under that particular combination of feature bins (# of sterilizing granulomas with cytokine A at level X and cytokine B at level Y out of total # of granulomas with cytokine A at level X and cytokine B at level Y). For each pairwise cytokine comparison, the frequency of sterilization under that particular combination of cytokines and levels is plotted and summarized in heat-map form. White squares indicate that granulomas with particular combination of cytokines at those levels did not occur.

To further evaluate the direct relationship between T cell cytokines and granuloma sterilization, we evaluated the effects of pairwise-cytokine combinations and magnitude of response on the frequency of sterilization using Matlab. First, for each granuloma (N = 133, only from animals necropsied after 17 weeks post-infection), T cell cytokines were binned according to the quartile distributions. Binned data provide the ability to evaluate the combinatorial trends without specific focus on absolute percentages. Briefly, for each pair of cytokines, for example IFN-γ and IL-2 across all granulomas, quartiles were calculated (Fig. 7A). The continuous percentages were transformed into discrete bins representing bin 1 (quartile 1, 0–25^th^ percentile), bin 2 (quartile 2, 25^th^–50^th^), bin 3 (quartile 3, 50^th^–75^th^), and bin 4 (quartile 4, 75^th^–100) of cytokine responses, as described in Fig. 7A. Numbers of granulomas in each bin were counted and summarized in a 4×4 co-occurrence matrix. Frequencies at which each of the pairwise combination occurred in the total number of granulomas were also calculated and plotted (Fig. 7A & B) as a density heat map (Fig. 7B) ranging from low represented by “dark blue” to high by “dark red”. Even though there were 133 granulomas included in this analysis, not all bins have equal representative number of granulomas, due to the variation in the magnitudes of response. However, the most commonly occurring pair of cytokines was IFN-γ in combination with IL-2 and TNF, and IL-10 with IL-2, IL-17 and TNF ranging from low to high magnitude. These heat maps confirm the correlation data detailed above, including the high frequency of granulomas with pro- and anti-inflammatory T cells.

Next, to evaluate the combinatorial effect of different cytokine combinations and magnitudes on the ability to attain bacterial containment, we calculated the frequency with which sterilization occurred under different cytokine combinations (Fig. 7C). Sterilization frequency matrices were constructed in a similar fashion as described above by calculating the number of times sterilization of a granuloma was observed (e.g. 0 CFU) given in a particular combination of cytokines and frequencies in granulomas. For example, there were 5 granulomas binned at bin 3/3 for IL-2 and IFN-γ (Fig. 7A) cytokine combinations respectively, of which 4 granulomas were sterile and 1 grew Mtb (non-sterile). Therefore the sterilization frequency for bin 3/3 is 80%, and represented at the respective density (color) heat map for 80% (Fig. 7C.a). These resulting density heat maps allowed us to visualize which conditions are associated with the highest frequency of sterilization. This approach revealed interesting features relating to bacterial containment and T cell cytokines.

For a large majority of cytokine combinations, there was no apparent direct relationship between cytokine combinations, magnitudes and bacterial control. However, the notable exceptions are the combinations of IL-10 with IL-2 (Fig. 7C.e) or IL-17 (Fig. 7C.h) or TNF (Fig. 7C.i), which had highest rates of sterilization, with frequencies >70%. Interestingly, T cells producing higher frequencies of IL-17 and IL-10 in the same granuloma give rise to high rates of sterilization, supporting our findings described in Table 1. A similar trend was also observed for IL-10 and TNF, whereas the more traditional T-1 responses (e.g. high frequencies of IFN-γ, IL-2 or TNF in the same granuloma) were not strongly associated with sterilization. These results reinforce that the balance of pro-inflammatory and anti-inflammatory cytokine responses is important for bacterial containment in the granuloma.

### Granuloma T cell profiles overlap amongst different clinical states

Our primary goal was to investigate and understand the dynamics of T cell function within the spectrum of Mtb lung granulomas irrespective of the clinical status of the animal. Nonetheless, to address whether clinical state was important in the T cell responses observed, we further analyzed the granuloma T cell profiles based on clinical status (active disease, latent infection, and 11 weeks post-infection). Overall, as with the bacterial burden, there was substantial overlap in cell profiles and T cell cytokine responses of granulomas amongst all clinical classifications. Animals infected for ∼11 weeks presented with granulomas having total cell numbers similar to those of active disease. However, the median total cell numbers were significantly greater in granulomas from monkeys with active disease (p = 0.027, Dunn’s multiple comparison test) when compared to those with latent infection (S5A Fig.). Similarly, granulomas from animals with active disease had significantly higher CD3+ T cell counts and frequency of CD3+ T cells when compared to granulomas from animals with latent infection (S5B–S5C Fig.). Granulomas from animals infected for ∼11weeks had the highest T cell counts compared to all other animals (p<0.0001) (S5B–S5C Fig.), suggesting that early in infection, the T cell response in granulomas is more robust.

**Figure 8:**
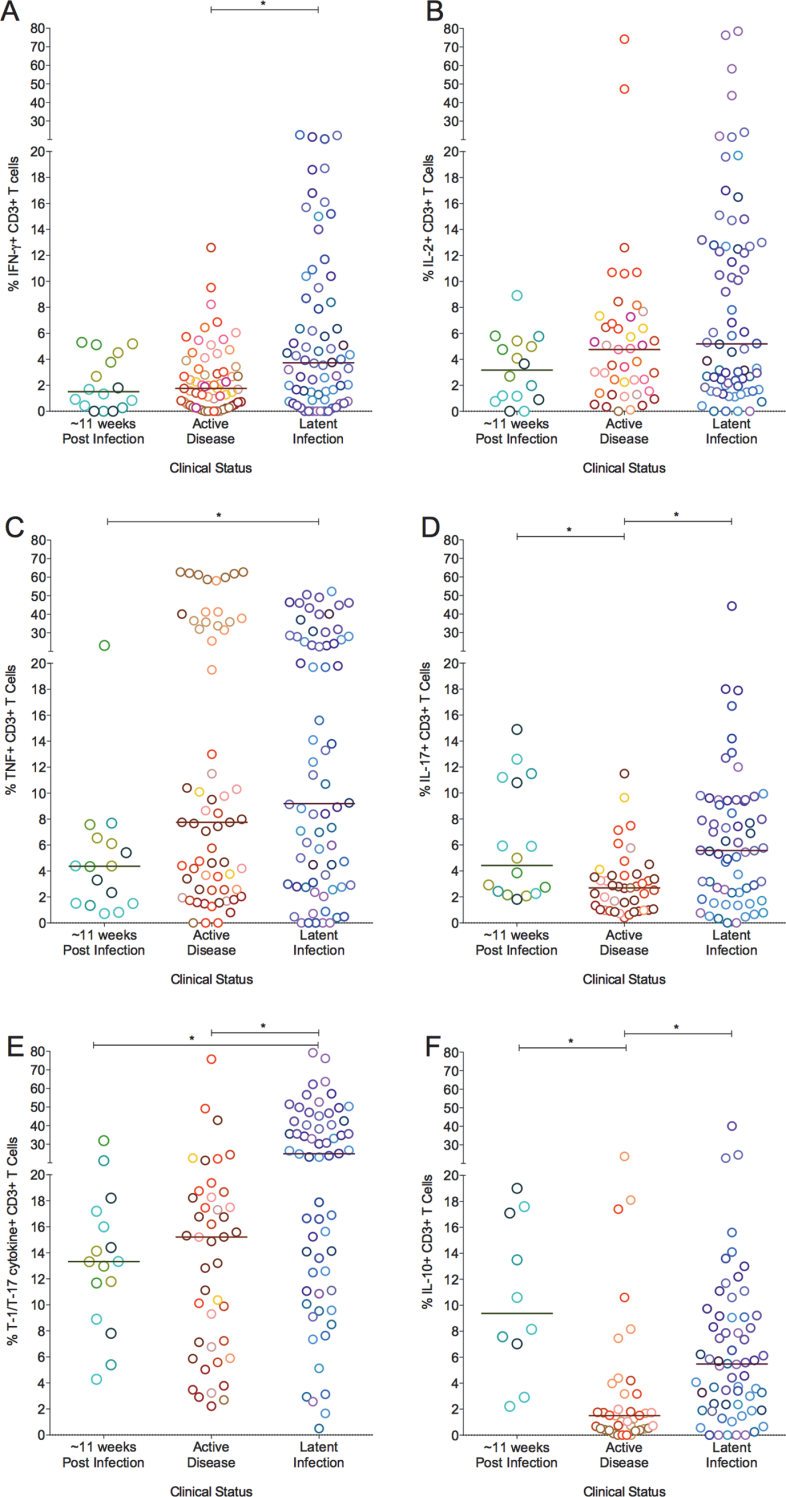
The proportion of T cells with cytokine response of IFN-γ (A), IL-2 (B), TNF (C), IL-17 (D), T-1/T-17 (E) and IL-10 (F). Each symbol represents a granuloma. Each color represents an animal. Granulomas are grouped based on the clinical status of the animals. Solid line indicates median response. (*: p<0.05; **: p<0.001, ***: p<0.0001. Dunn’s multiple test comparison).

Frequencies of cytokine-producing T cells in granulomas also overlapped across monkeys of different clinical classifications (Fig. 8A–F), however, there were some modestly distinguishing features. The frequency of IFN-γ and IL-17 producing T cells was modestly (but significantly) higher in granulomas from animals with latent infection, compared to granulomas from active disease animals. Granulomas from animals with latent infection had significantly higher frequencies of T-1/T-17 (p = 0.0012) T cells than those from animals with active disease and those infected for ∼11 weeks. The frequencies of IL-10 cytokine producing T cells were significantly lower in granulomas from animals with active disease (1.5%) compared to those infected for ∼11 weeks (9.3%) or with latent infection (5.5%) (Fig. 8F). Nonetheless, the responses in individual granulomas from animals of all infection outcome classifications were variable, with both high and low responding granulomas seen in most animals.

We assessed correlation amongst cytokine responses of individual granulomas to understand the relationship of T cell function in different clinical categories. There was a significant negative correlation between IL-2 and IL-10 exclusively (S2 Table) in those animals infected for ∼11 weeks. Granulomas from animals with active TB demonstrated a significant positive correlation between T cells producing IFN-γ and those producing IL-10 in addition to T cells producing pro-inflammatory cytokines. However, granulomas from animals with latent infection had a significant direct correlation between multiple pro- and anti-inflammatory cytokines (S2 Table). When we analyzed the association between T cell cytokines and bacterial burden, granulomas that had the highest bacterial burden in individual lesions from animals infected for ∼11 weeks had a negative correlation between IL-10 and bacterial burden, suggesting that IL-10 might also play a role in the early establishment of bacterial control in the granulomas (S3 Table).

### Systemic T cell responses do not reflect local responses

Studies in humans to understand immune responses during the course of Mtb infection, drug treatment, or vaccine testing rely primarily on analysis of systemic (blood) T cell responses. There is a significant knowledge gap as to how systemic responses relate to local responses in the lung, particularly at the granuloma level. This study provided an opportunity to perform preliminary evaluation of the relationship of T cell cytokine responses between the peripheral blood (systemic compartment) and granulomas (local compartment). To evaluate the complex dataset (comparing one measure of blood data to multiple measures of granuloma data from an animal), we used a simple mathematical equation to calculate Euclidean distances.

**Figure 9:**
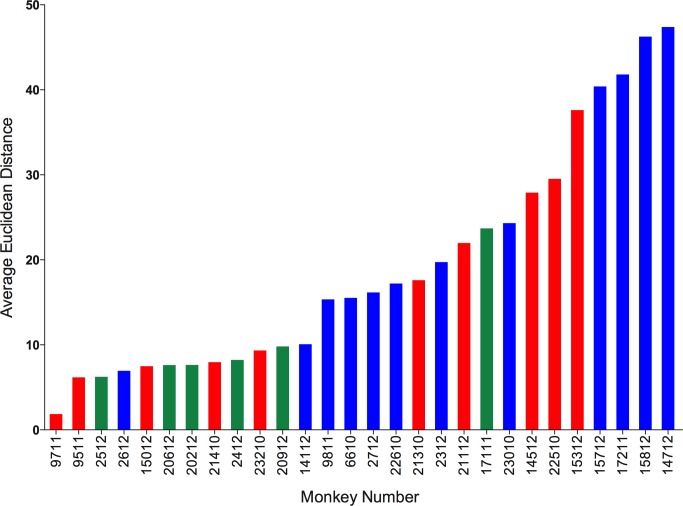
Systemic and local T cell responses. Euclidean distance between T cell cytokines from blood (systemic) and each of the individual granulomas (local) of animal were calculated. The distances were averaged per animal and represented as the average Euclidean distance. Average Euclidean distance provides an estimate for “relatedness” between the systemic T cell response and local T cell response. Each bar represents an animal. Animals with active disease are represented in red, latent infection in blue and those infected for ∼11 weeks in green. Animals are arranged in increasing average Euclidean distance.

We calculated Euclidean distances on datasets for which complete cytokine data were available for blood (PBMC) and granulomas (N = 120 granulomas and 28 animals). We calculated the Euclidean distance between the blood of an animal and all lesions from that animal. We then calculated the average distance for each animal. This provides an estimate for “relatedness” in terms of distance between the systemic and local T cell responses (i.e., smaller distance means closer or more similar the T cell responses, while larger distance means further away or more dissimilar the T cell responses between blood and granulomas are). Surprisingly, we observed a range of distances ranging from 2 to 48 (Fig. 9). This suggests, that for some animals, systemic T cell responses can reflect and are a reasonably good estimate of the local T cell response. While, for other (most) animals, systemic T cell responses are very different from local T cell responses and therefore do not reflect the local T cell responses accurately. The Euclidean distance between blood and granuloma responses was not related to disease state, as animals that are 11 weeks post-infection, active and latent infection were found all along the spectrum of responses.

We further explored the factors that could be associated with the variation in the T cell responses between systemic and local compartments. We correlated the average distance between systemic and local response with overall bacterial burden (CFU score), gross pathology (pathology score) and total number of lung granulomas of the animal. There was no significant correlation observed with either bacterial burden or the pathology score of the animal. However, there was significant correlation between the average distance and the total number of lung granulomas (lesions) of the animal (Spearman ρ 0.4046, Prob>| ρ| 0.0327) (S6 Fig.), (i.e., the greater the number of lung granulomas, the larger the difference in T cell responses between blood and granuloma). This further suggests the existence of spectrum of granulomas within an animal affects the systemic T cell responses. This has important implications for the search for blood biomarkers.

Next, we investigated whether the average T cell granuloma cytokine response of an animal correlated with its systemic (blood) cytokine response. We performed a multivariate analysis, and used non-parametric Spearman’s ρ for correlations. In animals with active disease and latent infection, overall there was no correlation observed with the exception of a significant direct correlation between local and systemic TNF producing T cells (S4 Table). When analyzed according to the clinical status of the animals, those with active disease had significant direct correlation between local and systemic responses for TNF and IL-17 producing T cells, while there was no correlation observed in animals with latent infection. Similarly, in animals infected for ∼11 weeks, there was a significant negative correlation (S4 Table) between blood and average granuloma responses for IL-17 and IL-10 producing T cells. These findings suggest that the systemic T cell responses do not accurately reflect the local (lung) T cell responses.

## Discussion

T cells are a major cellular component of tuberculosis lung granulomas and are known to play an important role in containment and progression of Mtb infection. Yet, there are many unanswered questions regarding the functional characteristics of T cells within granulomas, including the relationship between T cell cytokine responses and bacterial burden at the local level, and how T cell systemic responses (in the blood) relate to the T cell responses in granulomas. Much of what we know about T cell function in tuberculous lungs comes from mice, which do not develop the full spectrum of granulomas seen in human tuberculosis. Furthermore, functional data assessing T cell activity within individual granulomas in clinically active disease and latent infection are lacking. To address these questions, we used the cynomolgus macaque model of TB, a non-human primate model that recapitulates key hallmarks of human TB [26].

Our primary goal was to investigate and understand the dynamics of T cell function within the spectrum of Mtb lung granulomas. We show here that each granuloma within a single host is independent with respect to total cell numbers, frequency of T cells, the pattern of cytokine profile, and bacterial burden. We observed considerable overlap within these components amongst various clinical states of the animals. This study and a recent publication from our lab [2] support that individual granulomas themselves are unique representations of infection state and cannot be classified as “active” or “latent”. Conventional clinical classifications of active disease and latent infection states are thus more suited for a global or “whole host” classification to reflect overall host status on infection and pathology. These findings are further supported by the radiological and histological studies from our and other laboratories, which demonstrate the highly dynamic and variable nature of granulomas during Mtb infection, establishing that each granuloma is unique even within the same animal [2,25,27,29,51–53]. Individual animals have a full spectrum of lesions varying from progressive granulomas with high bacterial burden to healed sterile granulomas, each with varying proportions of functional T cells producing both pro- and anti-inflammatory cytokines. Our current data provide further evidence to support the concept that a spectrum of Mtb infection not only exists amongst animals [52,54,55], but also within an individual animal where granulomas are independent of each other with varying magnitudes of bacterial numbers and host responses.

T cells secreting IFN-γ, TNF and IL-17 are generally assumed to be necessary for activation of macrophages and initiation of antimicrobial activity [12,14,56]. Due to the recruitment of activated T cells to the site of disease, T cell responses are also considered to be enriched at the site of disease (i.e. the granuloma) compared to the periphery (blood). These concepts are supported by studies from small animal models where whole lung homogenates were studied, and from studies using cells from pleural TB [57], bronchoalveolar lavage (BAL) samples from active TB patients [31,45,58,59] and non-human primates [60]. Our study supports the notion that cells are continuously recruited to the non-sterile granulomas, resulting in increased total cell count, T cell number and increase size of the granuloma, while the sterile granulomas are maintained with the minimum required cell numbers for the continued maintenance. Although our studies support a higher proportion of T cells in granulomas that produce cytokines following stimulation with Mtb-specific peptides in granulomas as compared to blood, a striking finding from the current study is that only a limited proportion of T cells in granulomas were making any of the 5 major cytokines chosen for analysis, irrespective of clinical disease status. The average frequency of T cells producing any of these cytokines was about 8%.

There are several possibilities for the unexpected low frequency of T cells observed in the granulomas. It is possible that re-stimulation with a limited number of antigen peptide pools simply does not represent the full T cell recognition of Mtb antigens. We think this is unlikely, since granuloma homogenates likely contains large numbers of Mtb antigens, and our stimulation assays were carried out in granuloma homogenates. Analysis of granulomas that were not restimulated with ESAT-6/CFP-10 peptides showed only slightly lower frequencies of T cell responses, supporting the notion that antigens are present in the homogenate. In addition, stimulation with Mtb-infected dendritic cells, which would present more Mtb antigens, did not yield a higher frequency of T cells expressing cytokines. Separate analysis of T cells from granulomas with multiple antigen peptide pools and analyzed by IFN-γ ELISPOT (similar to our previous findings [24,25]) gave comparable or even lower frequencies of T cells producing IFN-γ, compared to our ICS analysis. Thus, it is unlikely that the low frequency of responding T cells in granulomas is simply due to stimulation with a limited number of Mtb antigens, or relative insensitivity of the ICS method. There may be inhibition of T cell function due to regulatory T cells or inhibitory cytokines. Preliminary data on a small subset of granulomas suggests that most of IL-10 producing T cells in the granulomas are not Foxp3+Tregs (S7 Fig.), however, larger studies are needed to address the effect of regulatory T cells on other cytokines in granulomas. Another possibility is that Mtb itself might be down-regulating of T cell activity, as suggested in the literature [61,62]. Further evaluation of bacterial or host factors on T cell function at the local granuloma level is certainly warranted to address this possibility.

T cell exhaustion in granulomas is also another possible explanation for the low frequency of T cell responses. In chronic infections, including TB, T cells can become exhausted or down-regulated [39–41]. However, in most granulomas, non-specific stimulation of T cells resulted in an average of 50% of the cells capable of producing T-1/T-17 cytokines, suggesting that the T cells were not exhausted. Our limited data on T cell exhaustion markers (CTLA-4 and PD-1) did not support an inverse correlation with the cytokine response from granulomas. Nonetheless, this warrants further investigation. In addition to the very limited numbers of cells obtained from individual granulomas, tetramers for use in “Chinese” cynomolgus macaques are not currently available, which currently precludes a more thorough investigation of exhaustion of antigen specific T cells. It remains a distinct possibility that many of the T cells in non-human primate Mtb granulomas are not specific for Mtb antigens as described in mice [39], but are simply recruited to the granuloma due to inflammatory signals.

T cell responses in blood and lung tissues are complex. Recently, Nikitina, et al., showed that increased proportion of IFN-γ produced by effector T cell within lung tissues and blood is associated with increased lung pathology in humans [63], while Theron, et al., showed that there was no correlation in either Mtb-specific and non-specific IFN-γ responses in a high TB burden setting [41]. Current TB literature strongly suggests an association between high bacterial burden, and the poly-functional T cell response in the periphery [12,32,45,48,64,65]. In contrast, we demonstrated an inverse correlation between bacterial burden and total pro-inflammatory cytokine responses by T cells in granulomas. However, very few T cells in granulomas were poly-functional, even with re-stimulation. Our data support that higher frequencies of overall responding T cells are associated with fewer bacteria within a granuloma. Although most individual T cells appear to primarily produce a single cytokine, the granuloma itself is “poly-functional” since T cells producing T-1, T-17 or IL-10 cytokines are significantly correlated amongst each other within granulomas, and therefore must co-exist within the same granuloma. In our study, granulomas that had a higher proportion of T cells producing IL-10 in combination with T cells producing pro-inflammatory cytokines IL-2, TNF or IL-17 were associated with sterilization. Further, our data provide evidence for the co-existence of pro- and anti-inflammatory T cells in granulomas, in both sterile and non-sterile granulomas and in animals with active disease or latent infection. This supports the idea that a balance of inflammatory mediators at the individual granuloma level may contribute to the ability of granulomas to both kill bacteria [2] and limit pathology [10,12,36–38].

Assessing immune responses as a biomarker in human Mtb infection and in vaccine studies in humans relies heavily on sampling of blood [66–68]. However, there is no clear understanding of the relationship between systemic and local responses. In fact, our data support that the systemic responses do not accurately reflect local responses in granulomas. This relationship might be further complicated due to the existence of a spectrum at both local and systemic levels in macaques and humans. Even though BAL is considered to be a closer approximation of the lung, airway T cell responses differ from granulomas responses [24], and lung granulomas provide us with exact measures of local responses at the site of bacterial interactions with the host. Thus, caution should be used in interpreting and extrapolating data from peripheral T cell responses in humans, although that does not exclude the potential for biomarkers of risk to be determined in the blood.

The major limitation of our study is the paucity of cells from individual granulomas and the size of the multi-parametric flow cytometric panel. Although we started with more than 300 granulomas and obtained usable data from ∼150 granulomas for this manuscript, the unexpectedly high heterogeneity observed in the T cell responses in granulomas suggests that this is relatively small data set and more samples are necessary for further robust analyses and modeling. Therefore, the statistically significant differences in the responses reported on single cytokine responses between sterile and non-sterile responses here are only modest. Due to the low numbers of T cells in individual granulomas, which can be quite small in size [2], individual CD4+ and CD8+ T cell cytokine responses were not analyzed. Variable number of granulomas from animals could be considered as a potential bias. Even though this is a limitation of the study design, the major focus of this manuscript is the analysis based on individual granulomas irrespective of animals, and therefore it does not affect the outcome of analysis. Another limitation of this study is the lack of evaluation of Th2- type T cell responses at the site of disease. This is largely due to the restricted number of flow cytometry channels that were available for use, and difficulty in detecting IL-4 responses in our preliminary studies. Finally, only a pairwise-combinatorial cytokine effect on sterilization was performed, so that 133 granulomas were included in the analysis. A more complex combinatorial analysis requires larger numbers of granulomas. Clearly there is much more work to be done in this area, and we expect that further work will uncover additional factors that contribute to sterilization of granulomas.

In summary, we find that a range of granuloma T cell responses exists within an individual animal. Surprisingly, only limited numbers of T cells produce cytokines at the site of disease, which nonetheless were still able to control bacterial burden given the inverse correlation with the number of recoverable bacteria from the granuloma. Our findings provide further evidence for the importance of the balance between pro- and anti-inflammatory cytokines at the granuloma level for control of bacterial burden. Finally, the systemic responses do not generally reflect the local responses, which have considerable implications in terms of biomarker discovery and interpretation and provides insights into the functioning of T cells within granulomas.

## Methods

### Ethics statement

All experimental manipulations, protocols, and care of the animals were approved by the University of Pittsburgh School of Medicine Institutional Animal Care and Use Committee (IACUC). The protocol assurance number for our IACUC is A3187-01. Our specific protocol approval numbers for this project are 13122689, 11090030, 1105870, 12060181, and 11110045. The IACUC adheres to national guidelines established in the Animal Welfare Act (7 U.S.C. Sections 2131–2159) and the Guide for the Care and Use of Laboratory Animals (8^th^ Edition) as mandated by the U.S. Public Health Service Policy.

All macaques used in this study were housed at the University of Pittsburgh in rooms with autonomously controlled temperature, humidity, and lighting. Animals were singly housed in caging at least 2 square meters apart that allowed visual and tactile contact with neighboring conspecifics. The macaques were fed twice daily with biscuits formulated for nonhuman primates, supplemented at least 4 days/week with large pieces of fresh fruits or vegetables. Animals had access to water *ad libitem*. Because our macaques were singly housed due to the infectious nature of these studies, an enhanced enrichment plan was designed and overseen by our nonhuman primate enrichment specialist. This plan has three components. First, species-specific behaviors are encouraged. All animals have access to toys and other manipulata, some of which will be filled with food treats (e.g. frozen fruit, peanut butter, etc.). These are rotated on a regular basis. Puzzle feeders foraging boards, and cardboard tubes containing small food items also are placed in the cage to stimulate foraging behaviors. Adjustable mirrors accessible to the animals stimulate interaction between animals. Second, routine interaction between humans and macaques are encouraged. These interactions occur daily and consist mainly of small food objects offered as enrichment and adhere to established safety protocols. Animal caretakers are encouraged to interact with the animals (by talking or with facial expressions) while performing tasks in the housing area. Routine procedures (e.g. feeding, cage cleaning, etc) are done on a strict schedule to allow the animals to acclimate to a routine daily schedule. Third, all macaques are provided with a variety of visual and auditory stimulation. Housing areas contain either radios or TV/video equipment that play cartoons or other formats designed for children for at least 3 hours each day. The videos and radios are rotated between animal rooms so that the same enrichment is not played repetitively for the same group of animals.

All animals are checked at least twice daily to assess appetite, attitude, activity level, hydration status, etc. Following *M. tuberculosis* infection, the animals are monitored closely for evidence of disease (e.g., anorexia, weight loss, tachypnea, dyspnea, coughing). Physical exams, including weights, are performed on a regular basis. Animals are sedated prior to all veterinary procedures (e.g. blood draws, etc.) using ketamine or other approved drugs. Regular PET/CT imaging is conducted on most of our macaques following infection and has proved very useful for monitoring disease progression. Our veterinary technicians monitor animals especially closely for any signs of pain or distress. If any are noted, appropriate supportive care (e.g. dietary supplementation, rehydration) and clinical treatments (analgesics) are given. Any animal considered to have advanced disease or intractable pain or distress from any cause is sedated with ketamine and then humanely euthanatized using sodium pentobarbital.

### Animals

Cynomolgus macaques (*Macaca fascicularis*), >4 years of age, (Valley Biosystems, Sacramento, CA) were housed within a Biosafety Level 3 (BSL-3) primate facility as previously described [17,24,25] and as above. Animals were infected with low dose *M. tuberculosis* (Erdman strain) via bronchoscopic instillation of about 25 colony-forming units (CFUs)/ monkey to the lower lung lobe. Infection was confirmed by tuberculin skin test conversion and/or lymphocyte proliferation assay six weeks post-infection [24]. Serial clinical, microbiologic, immunologic, and radiographic examinations were performed, as previously described. Based on defined clinical criteria, radiographic, and microbiologic assessments during the course of infection monkeys were classified as having latent infection or active disease 6–8 months after infection as described previously [25,26,29]. In addition, animals that were infected with Mtb and necropsied ≤11 weeks after infection were also included in this study.

### Necropsy

Necropsy was performed as previously described [17,24,25,29]. Briefly, an ^18^F-FDG PET-CT scan was performed on every animal 1–3 days prior to necropsy to measure disease progression and identify individual granulomas as described [29]. At necropsy, monkeys were maximally bled and humanely sacrificed using pentobarbital and phenytoin (Beuthanasia; Schering-Plough, Kenilworth, NJ). Individual lesions previously identified by PET-CT and those that were not seen on imaging from lung and mediastinal lymph nodes were obtained for histological analysis, bacterial burden, and immunological studies [29]. A veterinary pathologist described gross pathologic findings. To quantify gross pathologic disease (disease burden), a necropsy score was developed in which points were given for TB disease: number, size, and pattern of granulomas distributed in each lung lobe and mediastinal lymph node and in other organs each lung lobe, lymph node, and visceral organ were included and enumerated, and an overall score was determined as previously described [25]. The size of each granuloma was measured at necropsy and by pre necropsy scan [69]. Representative sections of each tissue were homogenized into single-cell suspensions for immunologic studies, flow cytometric analysis, and bacterial burden, as previously described [17,24,26,65].

### Bacterial burden

200μl of each granuloma homogenate were plated in serial dilutions onto 7H11 medium, and the CFU of *M. tuberculosis* growth were enumerated 21 days later to determine the number of bacilli in each granuloma [2,25]. As a quantitative measure of overall bacterial burden, a CFU score was derived from the summation of the log-transformed CFU/gram of each sample at the time of necropsy, as previously described [25].

### Flow cytometry

Flow cytometry was performed on a random sampling of granulomas (4–12 granulomas per animal). Although about 300 granulomas were initially analyzed, a cutoff of total number of T cells by flow cytometry was used to avoid introducing error due to analysis of very small T cell populations. Thus, a total of 149 granulomas from 34 animals were fully analyzed for this study. Single cell suspension of individual lung granulomas was stimulated with peptide pools of Mtb specific antigens ESAT-6 and CFP-10 (10μg/ml of every peptide) in the presence of Brefeldin A (Golgiplug: BD biosciences) for 3.5 hours at 37°C with 5% CO2. Positive control included stimulation with phorbol dibutyrate (PDBu) and ionomycin and an isotype control were included whenever additional cells were available. The cells were then stained for Viability marker (Invitrogen), surface and intracellular cytokine markers. Flow cytometry for cell surface markers for T cells included CD3 (clone SP34-2; BD Pharmingen), CD4 (clone L200; BD Horizon) and CD8 (clone SK1; BD biosciences). In addition, B cell marker CD20 (clone 2H7; eBioscience) and macrophage marker CD11b (clone Mac-1; BD Pharmingen) were included as the dump channel. Intracellular cytokine staining panel included pro-inflammatory cytokines: T-1 [IFN-γ (clone B27), IL-2 (clone MQ1-17H12), TNF (clone MAB11)], T-17 [IL-17 (clone eBio64CAP17) and anti-inflammatory (Regulatory) IL-10 (clone JES3-9D7) markers. In a subset of granulomas, exhaustion markers PD-1 (clone EH12.2H7, Biolegend) and CTLA-4 (Clone BN13, BD Pharmigen) were used to stain a subset of granulomas. Data acquisition was performed using a LSR II (BD) and analyzed using FlowJo Software v.9.7 (Treestar Inc, Ashland, OR). Supplementary figure 8 (S8 Fig.) describes the gating strategies employed for analysis. Supplementary figure 9 (S9 Fig.) provides detailed description of gating strategies in comparison with PBMC for clarity. Cytokine data presented in this manuscript are gated on CD3+ T cells.

### PBMC isolation

Heparinized blood was drawn from the animals prior to necropsy (terminal bleed). PBMCs were isolated via Percoll gradient centrifugation as previously described [70]. One million cells were stimulated with each of the antigens and controls, and incubated in similar conditions as described above for 6 hours. Stimulated PBMC were stained using the same panel of markers, acquired and analyzed as described above.

### Statistical analysis

D’Agostino & Pearson Omibus normality test was performed, on all data described in this manuscript. Since the data were not normally distributed, nonparametric t test was used when comparing two groups (Mann-Whitney test). Kruskal-Wallis test was used to compare more than two groups with post hoc analysis Dunn’s multiple test comparisons. *P* values ≤0.05 were considered significant. Statistical analysis was performed using GraphPad Prism v6 (GraphPad Software, San Diego, CA). For multivariate analysis, JMP Pro 10 (SAS) package was used. Nonparametric Spearman’s ρ was calculated for correlations (multivariate analysis) using JMP Pro v10 (SAS Institute Inc.).

Frequency of cytokine co-expression and sterilization in granulomas was implemented using Matlab (Mathworks, Natick, MA). Briefly, for each variable of T cell cytokine (IFN-γ, IL-2, TNF, IL-17 and IL-10) dataset, continuous values for each individual granuloma were binned into one of four categories depending on quartile distribution (bins 1, 2, 3, or 4) (Fig. 7A). We counted the number of times a particular combination of the variables occur and summarized these in a 4×4 co-occurrence matrices spanning all four bins comparing in a pairwise fashion across all the potential combinations. For each of the pairwise combination, the frequency of that occurrence is plotted and summarized in a heat map. Similarly, sterilization frequency matrices were constructed calculating the number of times sterilization occurred in a given combination of bins (number of sterilizing granulomas with a variable A at level X and variable B at level Y out of the total number of granulomas with variable A at level X and variable B at level Y). Each comparison was plotted as a heat map. For this analysis granulomas from animals with established clinical status (active disease or latent infection) were used (N = 113).

Euclidean distances were calculated in Microsoft Excel (for mac 2011), by utilizing the formula

$$\sqrt{{\left(GIFN\gamma -BIFN\gamma \right)}^{2}+{\left(GIL2-BIL2\right)}^{2}+{\left(GTNF-BTNF\right)}^{2}+{\left(GIL17-BIL17\right)}^{2}}$$

where suffix “G” represents T cell cytokine response from the granuloma (local) and “B” represents those from blood (systemic). Datasets for which complete cytokine data were available for individual granulomas (N = 120 granulomas) and blood (PBMC) (N = 28 animals) were used for this analysis. Distances were calculated for each granuloma with the T cell cytokine response of granuloma and blood of that animal. Average distances were then obtained by averaging the granuloma to blood distance for all the granulomas from a particular animal

## Supporting Information

S1 Fig Bacterial burden represented as log_10_ CFU per granuloma per animal. Each symbol is a granuloma, solid line indicates median log_10_ CFU per animal (of the granulomas included in this study). Greyed area represents sterile granulomas (log_10_ 1), which were assigned arbitrary range of values (log_10_ 0.1 and 0.99) to depict individual granulomas. Circles indicate non-sterile granulomas and squares indicate sterile granulomas. Clinical status is represented for each animal along with monkey number. Animals with active disease (A), latent infection (L) and those which are ∼11 post infection (E) had a spectrum of bacterial burden in granulomas, and sterile granulomas were present in each clinical groups. Animals are arranged in the order of increasing post-Mtb infection time.
(TIF)

S2 Fig Cytokine profile of individual granuloma per animal. The proportion of T cells with IL-2 (A), TNF (B), IL-17 (C), T-1/T-17 (D) and IL-10 (E) response from each animal. Each symbol represents a granuloma. Each color represents an animal. Granulomas are marked based on bacterial burden of the granuloma: non-sterile in open circles and sterile in open squares. Clinical status is represented for each animal along with monkey number [“E”: ∼11 weeks post infection; “A”: Active disease; “L”: Latent Infection]. Each individual granuloma had a distinct cytokine profile and there was a range of cytokine profile in an animal. Animals are arranged in the order of increasing post-Mtb infection time.
(TIF)

S3 Fig The proportion of T cells producing any or all of T-1 (IFN-γ, IL-2 and TNF) and T-17 (IL-17) cytokines in response to non-specific stimuli PDBu and ionomycin from a subset of individual granulomas. Each symbol represents individual granuloma. Line indicates median response.
(TIF)

S4 Fig In lung granulomas,T cells expressing CTLA-4^−^PD-1^+^ was most common (median 8.3%), compared to CTLA-4^+^PD-1^−^ or CTLA-4^+^PD-1^+^ (p<0.0001, Dunn’s multiple comparison test) (A). The proportion of cytokine producing T cells [IFN-γ (**B**), IL-2 (**C**), TNF (**D**), IL-17 (**F**) and IL-10 (**G**)] with or without co-expression of exhaustion markers from subset of individual granulomas in response to Mtb specific RD-1 encoded ESAT-6 and CFP-10. Each symbol represents individual granuloma, and each shape represents granulomas from one animal. Line indicates median response.
(TIF)

S5 Fig A Total number of cells obtained from granuloma. Granulomas with cell counts less than the detection limit were assigned 9×10^4^ before correcting for the dilution factor. Animals infected for ∼11 weeks and those with active disease (∼11 weeks: median 7.8×10^5,^ IQR 2.35×10^5^–1×10^6^; active: median 6×10^5^, IQR 1.8×10^5^–1.7×10^6^) had significantly higher cell numbers when compared to those with latent infection (median: 1.9×10^5^, IQR 1.8×10^5^–4×10^5^) (p = 0.027, Dunn’s multiple comparison test). **B**. Total number of T cell counts, defined by CD3+ per granuloma. **C** is the total frequency of live CD3+ cells per granulomas and was used to extrapolate the absolute of T cell count per granuloma (B) from the total granuloma cell count (A). Granulomas from animals infected for ∼11weeks (median 5.9×10^4^; IQR: 3.8 ×10^4^–2×10^5^) had significantly higher (p<0.0001, Dunn’s multiple test comparison) T cell counts compared to active disease (median 9×10^3^; IQR: 3.4×10^3^–9 ×10^4^) and latent infection (median 3.3×10^3^; IQR: 1.4×10^3^–8.5×10^3^). Similarly, granulomas from animals infected for ∼11weeks (median 9.9%; IQR: 4.4% −24.78) had significantly higher (p<0.0001, Dunn’s multiple test comparison) frequency of total CD3+ T cells compared to active disease (median 3.1%; IQR: 1.3%–7.0%) and latent infection (median 1.3%; IQR: 0.55%–2.3%) Each dot represents a granuloma. Each color represents an animal. Granulomas are grouped based on the clinical status of the animal. Solid line indicates median in each group. Dotted line in **A** indicates the detection limit (1×10^5^) before the correcting for the dilution factor. (***: p<0.0001, **: p = 0.001, *:p = 0.01, Kruskal-Wallis test & Dunn’s multiple test comparison).
(TIF)

S6 Fig Correlation of average Euclidean distance between system and local response and number of lung granulomas per animal. (TIF)

S7 Fig Proportion of T cells producing IL-10 in Foxp3+ (Treg) and Foxp3- population in a small subset of granulomas (n = 30), in response to either ESAT-6/CFP-10 (Green circles) or PDBu+Ionomycin (Orange Circles). (TIF)

S8 Fig Representative flow cytometry plots (15012_RLL-GranA, stimulated with P&I) outlining gating strategies employed in the analysis of granuloma T cells. Viable cells were negatively selected based on the absence of viability marker (**A**). CD11b was used as one of the dump channels (**B**). Lung granulomas have debris, and they appear on the Y-axis, which is gated out (excluded) (**B**) to obtain cleaner population for further evaluation. Lymphocytes were selected based on SSC and FSC (i.e., size and granularity)(**C**). CD3+ were gated on the lymphocyte population and defined as T cells (**D**) from which cytokine producing T cells (**E**) were gated. Arrow indicates sequence of gating.
(TIF)

S9 Fig Detailed description of FACS gating strategies for lung granuloma (S8 Figure) in comparison with PBMC. **A** is the Forward (FSC) and Side scatter (SSC) area profile of both lung granuloma and PBMC. **B** is the viable cell population gated by gating on cells with less fluorescence intensity using the Indo-violet live/dead stain. **C** is the FSC-A and SSC-A display of the viable cells from **B**. To obtain cleaner T cell population, CD11b was used as dump channel and CD11b negative cells (**D**) were selected for further analysis. In the example representative flow plot **D**, there is a large population on the Y-axis. In our samples, this “population” is mostly lung and granuloma debris. Therefore, this population was gated out as shown in S8 Fig. **D2** shows bi-exponentially transformed plot of **D** for clarity. **E** is the FSC-A and SSC-A gated of **D**, and shows the lymphocyte population of cells. **F** is CD3 positive T cells selected based on positive staining for APC-Cy7. The frequency of CD3+ T cells or the number of CD3+ T cells in this particular example did not differ whether or not the population of the Y-axis from **D** was included. The cytokines were gated entirely on this selected CD3 positive population.
(TIF)

S1 Table Correlation of total cell numbers, T cell counts and bacterial burden per granuloma with granuloma size. (DOCX)

S2 Table Pairwise correlation of cytokine levels within granulomas based on clinical states. (DOCX)

S3 Table Correlation of bacterial burden and T cell cytokine response based on clinical states. (DOCX)

S4 Table Correlation of average T cell cytokine response of granuloma of an animal and its systemic response. (DOCX)
