# Supplementary figures and images for: Variability in Tuberculosis Granuloma T Cell Responses Exists, but a Balance of Pro- and Anti-inflammatory Cytokines Is Associated with Sterilization
Source: PLoS Pathog. 2015 Jan 22;11(1):e1004603. doi: 10.1371/journal.ppat.1004603 (PMC4303275; doi:10.1371/journal.ppat.1004603)

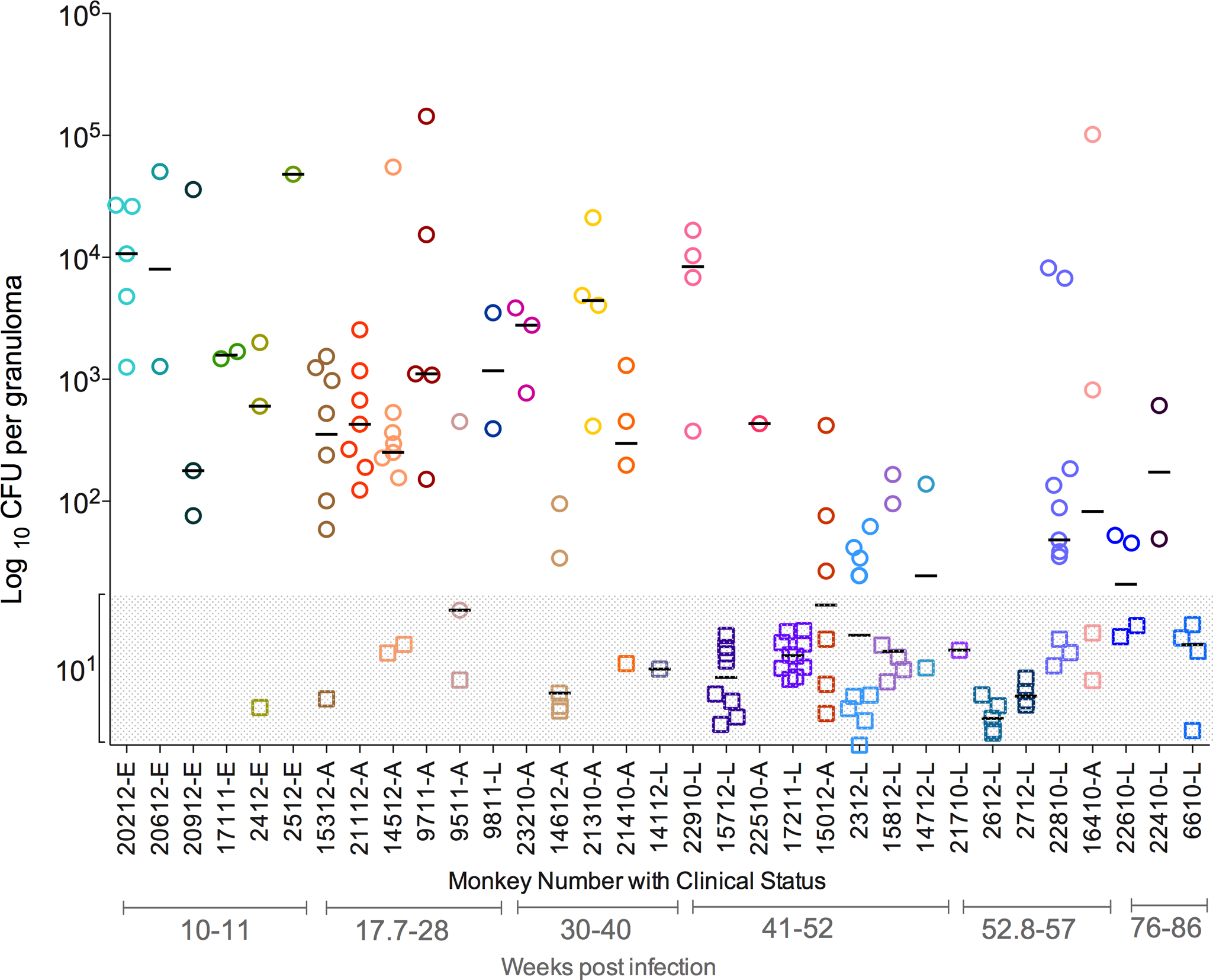

Supplement: S1 Fig — Each symbol is a granuloma, solid line indicates median log10 CFU per animal (of the granulomas included in this study). Greyed area represents sterile granulomas (log10 1), which were assigned arbitrary range of values (log10 0.1 and 0.99) to depict individual granulomas. Circles indicate non-sterile granulomas and squares indicate sterile granulomas. Clinical status is represented for each animal along with monkey number. Animals with active disease (A), latent infection (L) and those which are ∼11 post infection (E) had a spectrum of bacterial burden in granulomas, and sterile granulomas were present in each clinical groups. Animals are arranged in the order of increasing post-Mtb infection time. (TIF) [file ppat.1004603.s001.tif]

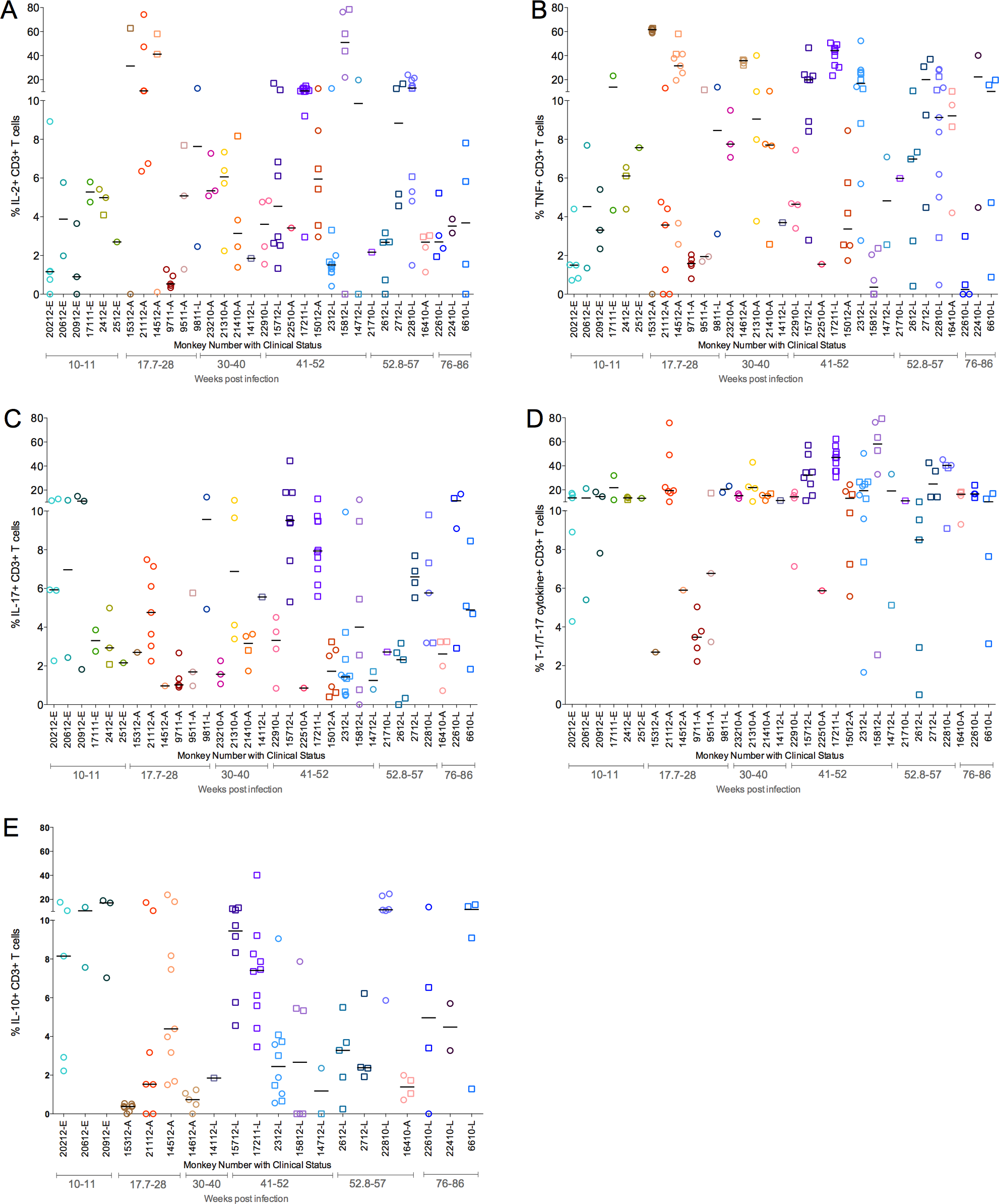

Supplement: S2 Fig — The proportion of T cells with IL-2 (A), TNF (B), IL-17 (C), T-1/T-17 (D) and IL-10 (E) response from each animal. Each symbol represents a granuloma. Each color represents an animal. Granulomas are marked based on bacterial burden of the granuloma: non-sterile in open circles and sterile in open squares. Clinical status is represented for each animal along with monkey number [“E”: ∼11 weeks post infection; “A”: Active disease; “L”: Latent Infection]. Each individual granuloma had a distinct cytokine profile and there was a range of cytokine profile in an animal. Animals are arranged in the order of increasing post-Mtb infection time. (TIF) [file ppat.1004603.s002.tif]

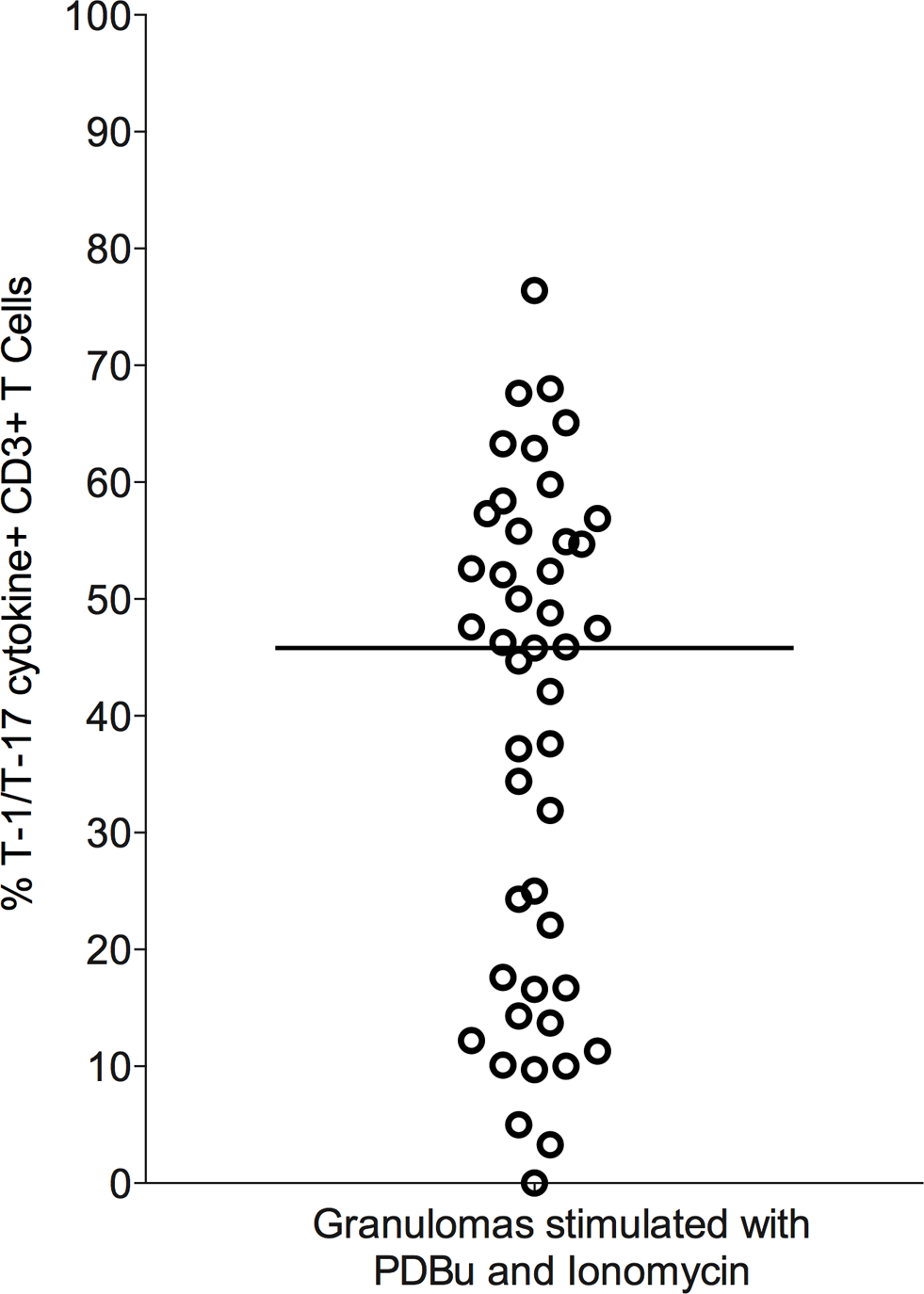

Supplement: S3 Fig — Each symbol represents individual granuloma. Line indicates median response. (TIF) [file ppat.1004603.s003.tif]

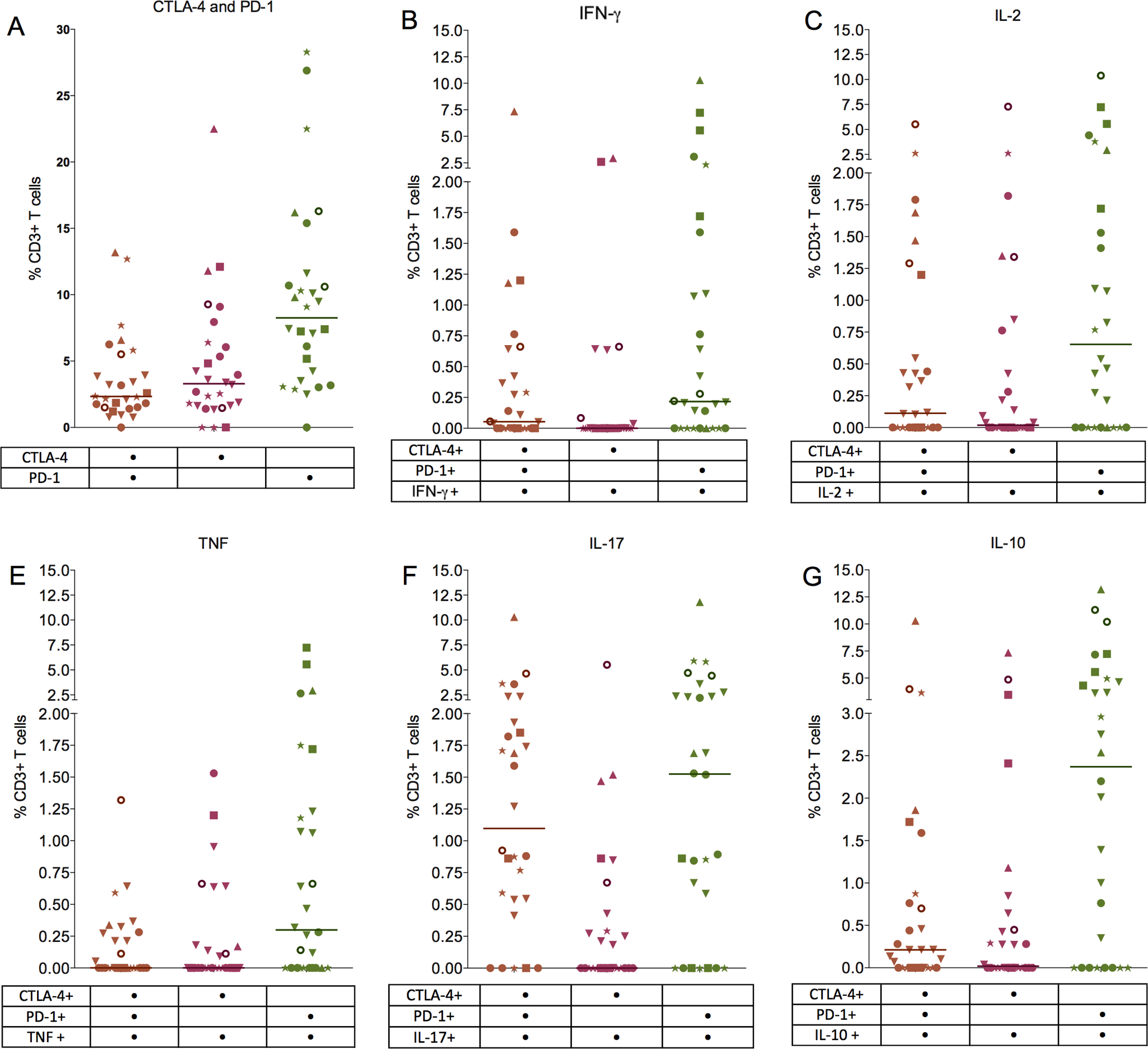

Supplement: S4 Fig — (A). The proportion of cytokine producing T cells [IFN-γ (B), IL-2 (C), TNF (D), IL-17 (F) and IL-10 (G)] with or without co-expression of exhaustion markers from subset of individual granulomas in response to Mtb specific RD-1 encoded ESAT-6 and CFP-10. Each symbol represents individual granuloma, and each shape represents granulomas from one animal. Line indicates median response. (TIF) [file ppat.1004603.s004.tif]

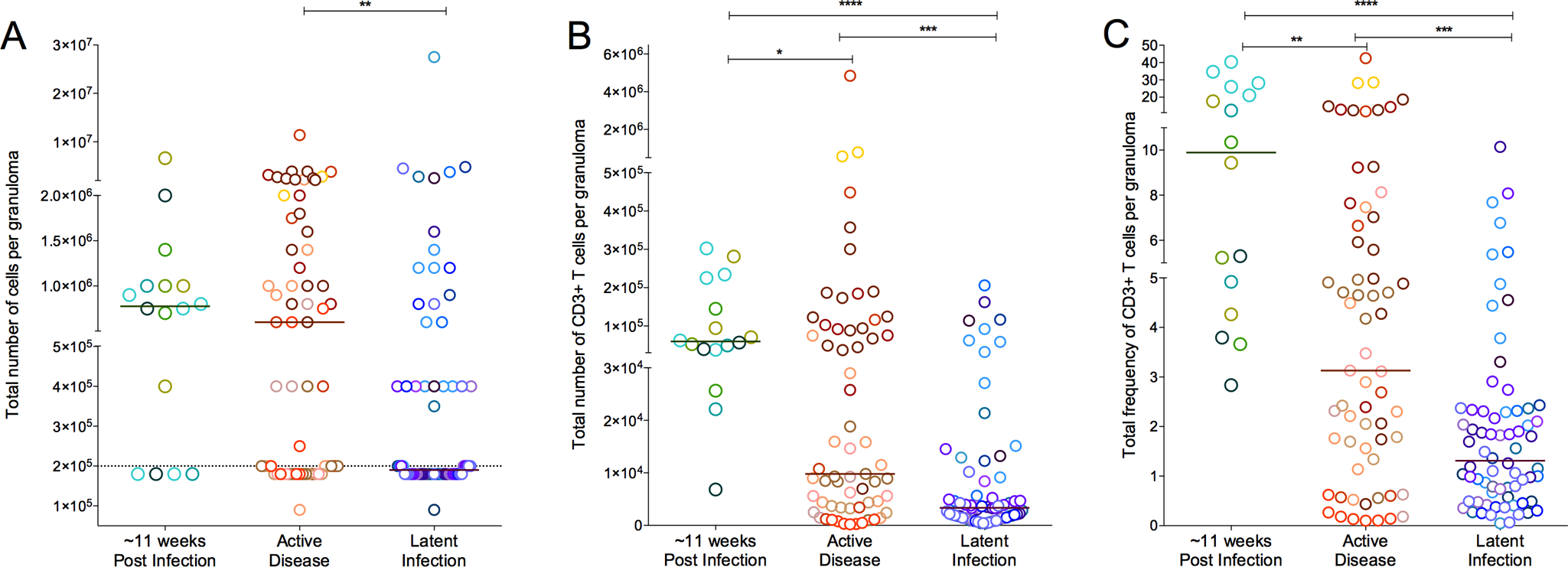

Supplement: S5 Fig — Granulomas with cell counts less than the detection limit were assigned 9×10^4^ before correcting for the dilution factor. Animals infected for ∼11 weeks and those with active disease (∼11 weeks: median 7.8×10^5^, IQR 2.35×10^5^–1×10^6^; active: median 6×10^5^, IQR 1.8×10^5^–1.7×10^6^) had significantly higher cell numbers when compared to those with latent infection (median: 1.9×10^5^, IQR 1.8×10^5^–4×10^5^) (p = 0.027, Dunn’s multiple comparison test). B. Total number of T cell counts, defined by CD3+ per granuloma. C is the total frequency of live CD3+ cells per granulomas and was used to extrapolate the absolute of T cell count per granuloma (B) from the total granuloma cell count (A). Granulomas from animals infected for ∼11weeks (median 5.9×10^4^; IQR: 3.8 ×10^4^–2×10^5^) had significantly higher (p<0.0001, Dunn’s multiple test comparison) T cell counts compared to active disease (median 9×10^3^; IQR: 3.4×10^3^–9 ×10^4^) and latent infection (median 3.3×10^3^; IQR: 1.4×10^3^–8.5×10^3^). Similarly, granulomas from animals infected for ∼11weeks (median 9.9%; IQR: 4.4% −24.78) had significantly higher (p<0.0001, Dunn’s multiple test comparison) frequency of total CD3+ T cells compared to active disease (median 3.1%; IQR: 1.3%–7.0%) and latent infection (median 1.3%; IQR: 0.55%–2.3%) Each dot represents a granuloma. Each color represents an animal. Granulomas are grouped based on the clinical status of the animal. Solid line indicates median in each group. Dotted line in A indicates the detection limit (1×10^5^) before the correcting for the dilution factor. (***: p<0.0001, **: p = 0.001, *:p = 0.01, Kruskal-Wallis test & Dunn’s multiple test comparison). (TIF) [file ppat.1004603.s005.tif]

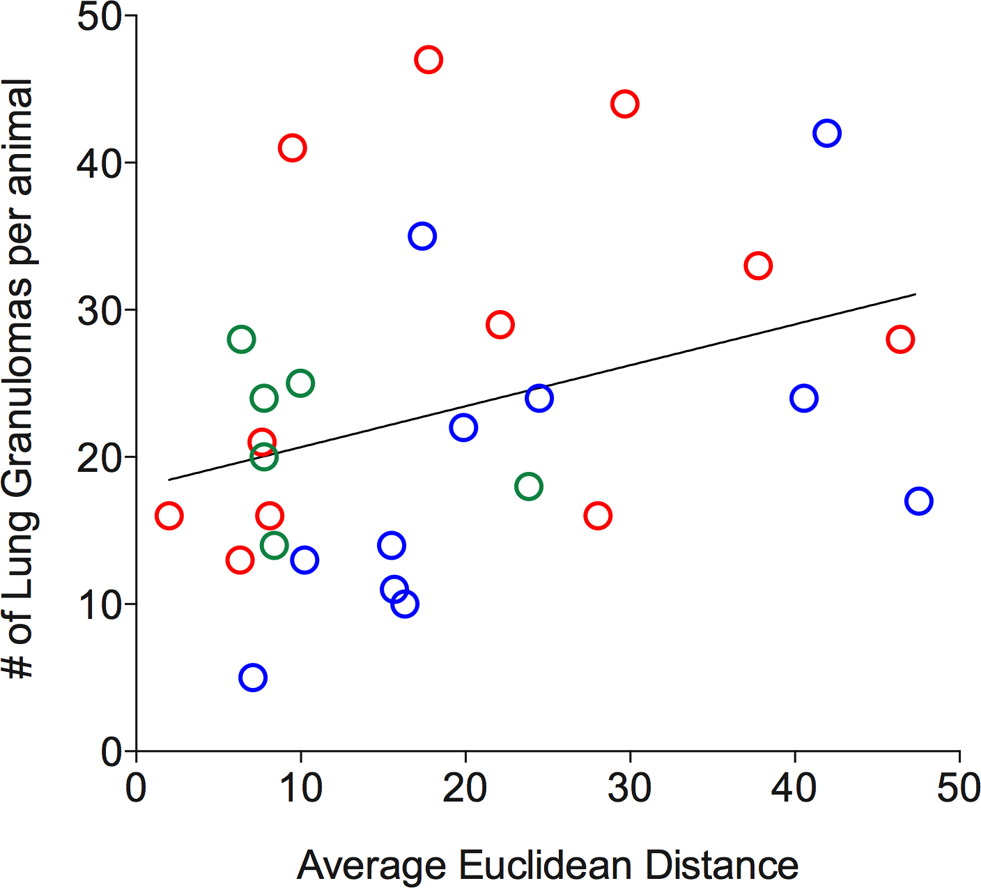

Supplement: S6 Fig — (TIF) [file ppat.1004603.s006.tif]

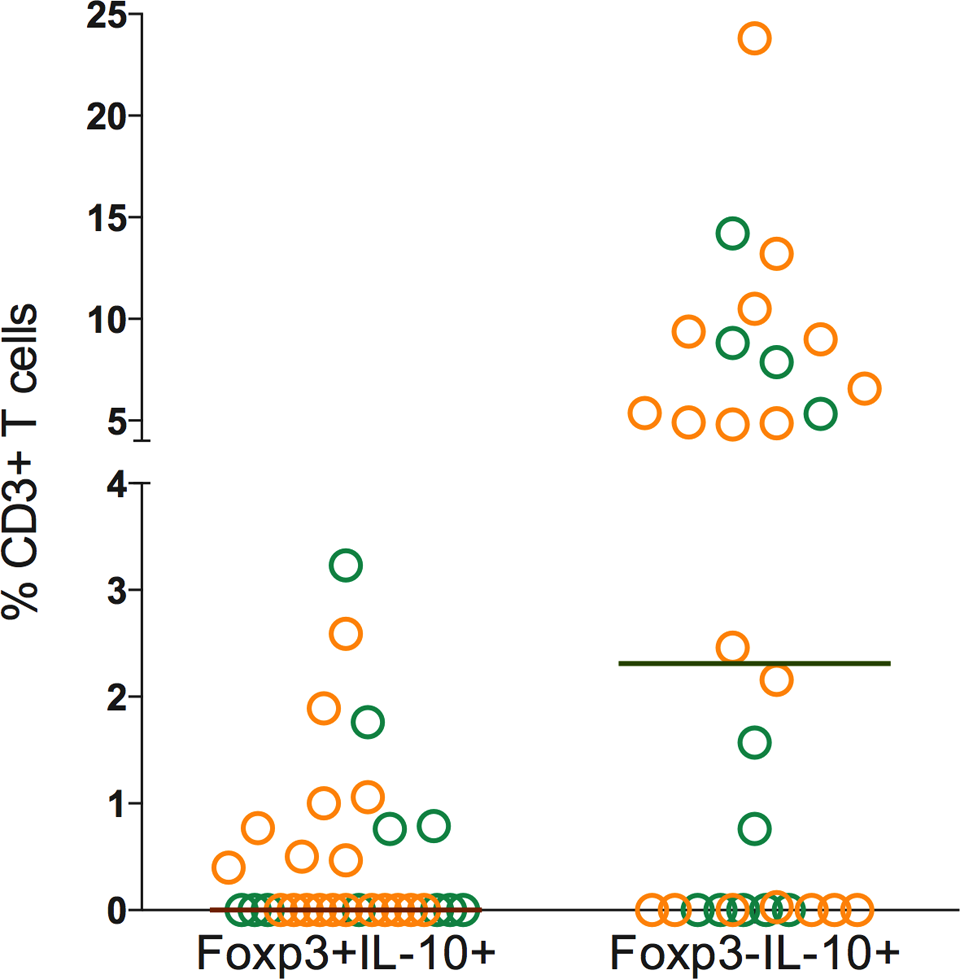

Supplement: S7 Fig — (TIF) [file ppat.1004603.s007.tif]

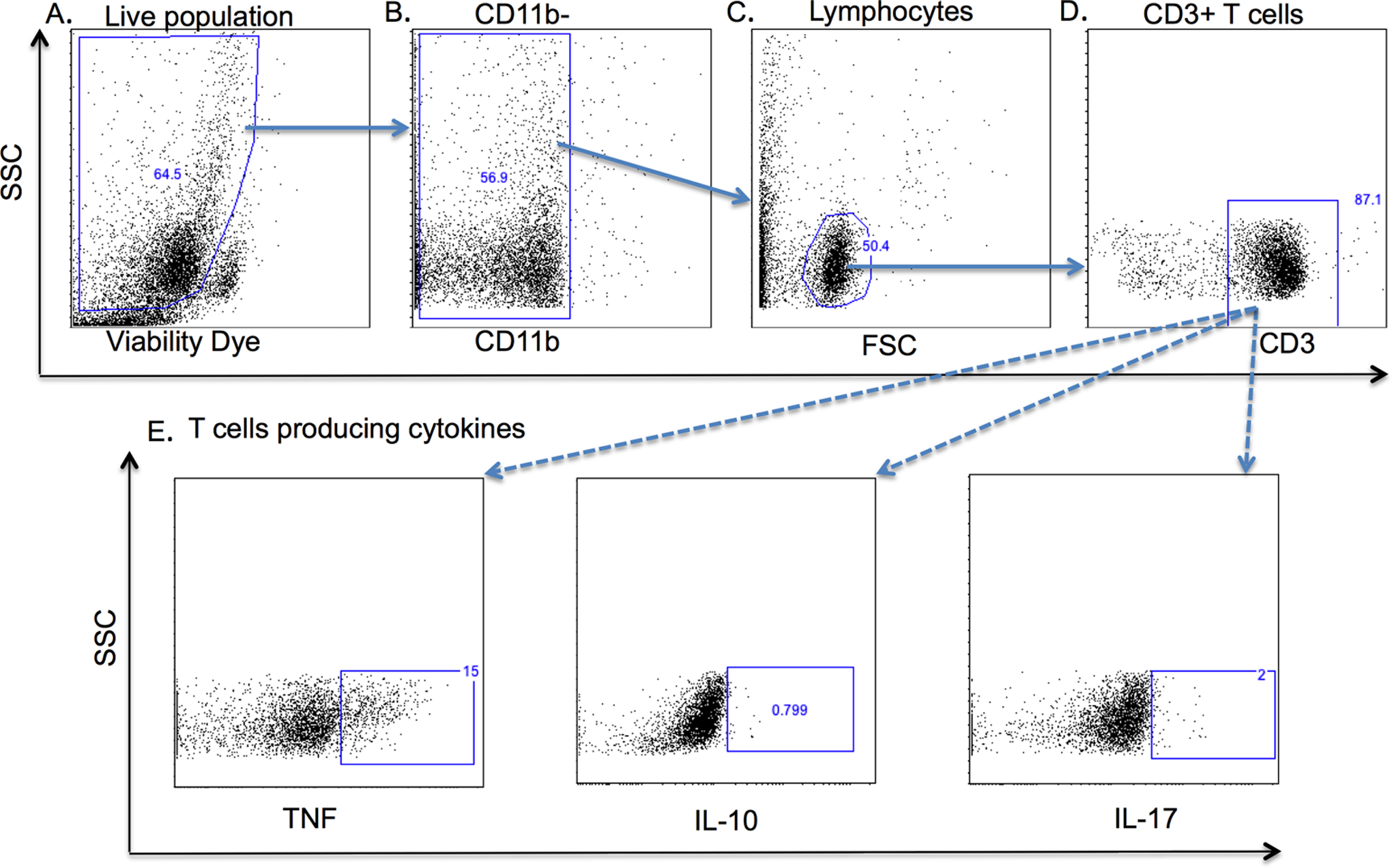

Supplement: S8 Fig — Viable cells were negatively selected based on the absence of viability marker (A). CD11b was used as one of the dump channels (B). Lung granulomas have debris, and they appear on the Y-axis, which is gated out (excluded) (B) to obtain cleaner population for further evaluation. Lymphocytes were selected based on SSC and FSC (i.e., size and granularity)(C). CD3+ were gated on the lymphocyte population and defined as T cells (D) from which cytokine producing T cells (E) were gated. Arrow indicates sequence of gating. (TIF) [file ppat.1004603.s008.tif]

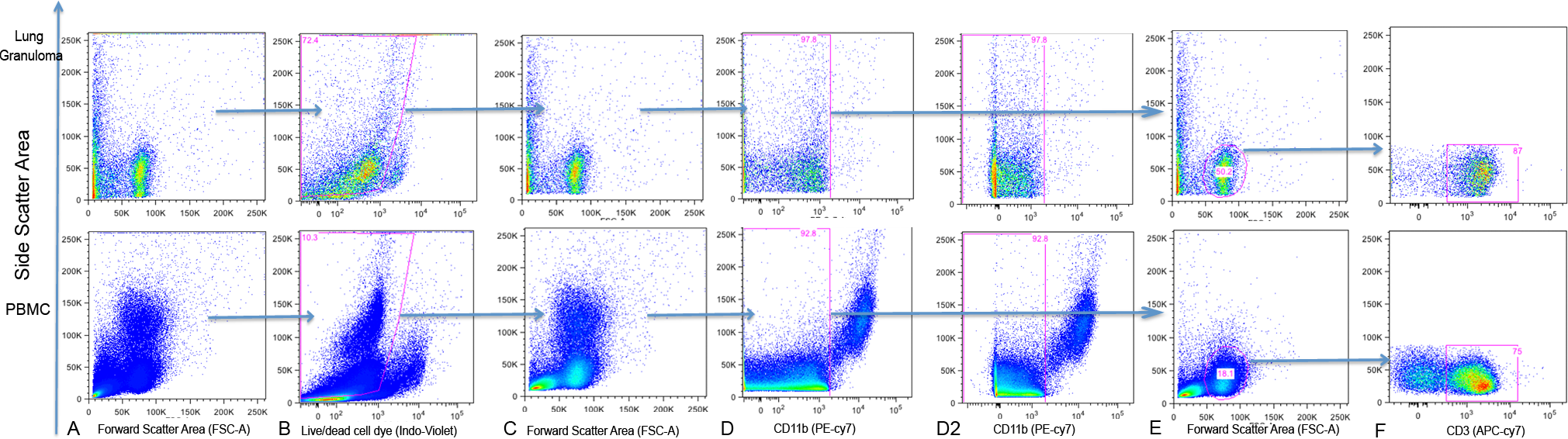

Supplement: S9 Fig — A is the Forward (FSC) and Side scatter (SSC) area profile of both lung granuloma and PBMC. B is the viable cell population gated by gating on cells with less fluorescence intensity using the Indo-violet live/dead stain. C is the FSC-A and SSC-A display of the viable cells from B. To obtain cleaner T cell population, CD11b was used as dump channel and CD11b negative cells (D) were selected for further analysis. In the example representative flow plot D, there is a large population on the Y-axis. In our samples, this “population” is mostly lung and granuloma debris. Therefore, this population was gated out as shown in S8 Fig. D2 shows bi-exponentially transformed plot of D for clarity. E is the FSC-A and SSC-A gated of D, and shows the lymphocyte population of cells. F is CD3 positive T cells selected based on positive staining for APC-Cy7. The frequency of CD3+ T cells or the number of CD3+ T cells in this particular example did not differ whether or not the population of the Y-axis from D was included. The cytokines were gated entirely on this selected CD3 positive population. (TIF) [file ppat.1004603.s009.tif]
