# Supplementary material for: Variability in Tuberculosis Granuloma T Cell Responses Exists, but a Balance of Pro- and Anti-inflammatory Cytokines Is Associated with Sterilization
Source: PLoS Pathog. 2015 Jan 22;11(1):e1004603. doi: 10.1371/journal.ppat.1004603 (PMC4303275; doi:10.1371/journal.ppat.1004603)
Supplement: S1 Table — (DOCX) [file ppat.1004603.s010.docx]

***Supplementary Tables***

**Table S1**

Correlation of total cell numbers, T cell counts and bacterial burden per granuloma with granuloma size.

|  | **Granuloma size vs. Total cell counts** | **Granuloma size vs. Total T cells** | **Granuloma size vs. Ratio of CD4:CD8** | **Granuloma size**  **vs. Log_10_ CFU/granuloma** |
| --- | --- | --- | --- | --- |
| **Spearman ρ** | **0.6523** | **0.5122** | **0.2769** | **0.7087** |
| **Prob>\|ρ\|** | **< 0.0001** | **< 0.0001** | **0.0086** | **< 0.0001** |
