# Supplementary material for: Variability in Tuberculosis Granuloma T Cell Responses Exists, but a Balance of Pro- and Anti-inflammatory Cytokines Is Associated with Sterilization
Source: PLoS Pathog. 2015 Jan 22;11(1):e1004603. doi: 10.1371/journal.ppat.1004603 (PMC4303275; doi:10.1371/journal.ppat.1004603)
Supplement: S2 Table — (DOCX) [file ppat.1004603.s011.docx]

**Table S2**

Pairwise correlation of cytokine levels within granulomas based on clinical states

| **Cytokine variables** | **~11 weeks post Infection** | | **Active Disease** | | **Latent Infection** | |
| --- | --- | --- | --- | --- | --- | --- |
|  | **Spearman ρ** | **Prob>\|ρ\|** | **Spearman ρ** | **Prob>\|ρ\|** | **Spearman ρ** | **Prob>\|ρ\|** |
| IL-2 vs IFN-**γ** | 0.4561 | 0.0758 | 0.2125 | 0.1712 | 0.0853 | 0.4826 |
| TNF vs IFN-**γ** | 0.4484 | 0.0815 | -0.1933 | 0.1291 | 0.0144 | 0.9055 |
| TNF vs IL-2 | 0.3414 | 0.1956 | 0.2403 | 0.1206 | 0.2839 | **0.0172** |
| TNF vs IL-10 | 0.7939 | **0.0061** | -0.2019 | 0.2177 | **0.3134** | **0.0131** |
| TNF vs IL-17 | 0.0412 | 0.8797 | **0.4737** | **0.0013** | **0.2763** | **0.0297** |
| IL-17 vs IFN-**γ** | -0.2419 | 0.3667 | **0.362** | **0.0171** | **0.3646** | **0.0036** |
| IL-17 vs IL-2 | -0.2958 | 0.266 | **0.5236** | **0.0003** | **0.3432** | **0.0063** |
| IL-17 vs IL-10 | **0.8303** | **0.0029** | 0.1262 | 0.6067 | **0.6818** | **<.0001** |
| IL-10 vs IFN-**γ** | -0.1411 | 0.6974 | **0.3918** | **0.0136** | **0.4285** | **0.0005** |
| IL-10 vs IL-2 | **-0.6991** | **0.0245** | 0.1835 | 0.4522 | **0.2831** | **0.0258** |
| T-1/T-17 vs IL-10 | **0.6485** | **0.0425** | 0.1741 | 0.476 | 0.255 | 0.0628 |
