# Supplementary material for: Variability in Tuberculosis Granuloma T Cell Responses Exists, but a Balance of Pro- and Anti-inflammatory Cytokines Is Associated with Sterilization
Source: PLoS Pathog. 2015 Jan 22;11(1):e1004603. doi: 10.1371/journal.ppat.1004603 (PMC4303275; doi:10.1371/journal.ppat.1004603)
Supplement: S3 Table — (DOCX) [file ppat.1004603.s012.docx]

**Table S3**

Correlation of bacterial burden and T cell cytokine response based on clinical states

| **Variables** | | **~11 weeks post Infection** | | **Active Disease** | | **Latent Infection** | |
| --- | --- | --- | --- | --- | --- | --- | --- |
|  |  | **Spearman ρ** | **Prob>\|ρ\|** | **Spearman ρ** | **Prob>\|ρ\|** | **Spearman ρ** | **Prob>\|ρ\|** |
| **Log_10_ CFU per granuloma** | **IFN**-**γ** | -0.1917 | 0.4768 | -0.0816 | 0.525 | 0.2154 | 0.0734 |
|  | **IL-2** | 0.0471 | 0.8625 | -0.2348 | 0.1296 | 0.1534 | 0.2048 |
|  | **TNF** | -0.3265 | 0.2172 | -0.2139 | 0.0923 | -0.1837 | 0.1279 |
|  | **IL-17** | -0.3794 | 0.1472 | 0.0334 | 0.8314 | -0.0689 | 0.5947 |
|  | **IL-10** | **-0.7212** | **0.0186** | 0.0203 | 0.9025 | 0.0651 | 0.6154 |
|  | **T-1/T-17** | -0.3971 | 0.1278 | -0.1687 | 0.2794 | 0.0563 | 0.664 |
