# Supplementary material for: Variability in Tuberculosis Granuloma T Cell Responses Exists, but a Balance of Pro- and Anti-inflammatory Cytokines Is Associated with Sterilization
Source: PLoS Pathog. 2015 Jan 22;11(1):e1004603. doi: 10.1371/journal.ppat.1004603 (PMC4303275; doi:10.1371/journal.ppat.1004603)
Supplement: S4 Table — (DOCX) [file ppat.1004603.s013.docx]

**Table S4**

Correlation of average T cell cytokine response of granuloma of an animal and its systemic response

| **Cytokine Variables** | **~ 11 weeks post infection** | | **Active disease and Latent infection** | | **Active Disease** | | **Latent Infection** | |
| --- | --- | --- | --- | --- | --- | --- | --- | --- |
|  | **Spearman ρ** | **Prob>\|ρ\|** | **Spearman ρ** | **Prob>\|ρ\|** | **Spearman ρ** | **Prob>\|ρ\|** | **Spearman ρ** | **Prob>\|ρ\|** |
| **IFN-γ** | 0.7143 | 0.1108 | 0.2017 | 0.3445 | 0.2909 | 0.3855 | 0.1868 | 0.5411 |
| **IL-2** | 0.1429 | 0.7872 | 0.3771 | 0.0761 | 0.4909 | 0.1497 | 0.1813 | 0.5533 |
| **TNF** | 0.3714 | 0.4685 | **0.553** | **0.0051** | **0.8455** | **0.001** | 0.2418 | 0.4262 |
| **IL-17** | **-0.8286** | **0.0416** | 0.3484 | 0.1121 | **0.7212** | **0.0186** | 0.1399 | 0.6646 |
